# The Proteasome-Family-Members-Based Prognostic Model Improves the Risk Classification for Adult Acute Myeloid Leukemia

**DOI:** 10.3390/biomedicines12092147

**Published:** 2024-09-22

**Authors:** Guangying Sheng, Jingfen Tao, Peng Jin, Yilu Li, Wen Jin, Kankan Wang

**Affiliations:** 1 Shanghai Institute of Hematology, State Key Laboratory of Medical Genomics, National Research Center for Translational Medicine at Shanghai, Ruijin Hospital, Shanghai Jiao Tong University School of Medicine, 197 Ruijin Er Rd., Shanghai 200025, China; shengesther@163.com (G.S.); taojingfen@foxmail.com (J.T.); jinpeng2339@163.com (P.J.); liyilu1183433617@163.com (Y.L.); jw11862@rjh.com.cn (W.J.); 2 Sino-French Research Center for Life Sciences and Genomics, Ruijin Hospital, Shanghai Jiao Tong University School of Medicine, 197 Ruijin Er Rd., Shanghai 200025, China; 3 School of Life Sciences and Biotechnology, Shanghai Jiao Tong University, 800 Dong Chuan Road, Shanghai 200240, China

**Keywords:** acute myeloid leukemia, proteasome family member, expression, overall survival, prognostic model, ELN stratification, therapy

## Abstract

**Background:** The accumulation of diverse molecular and cytogenetic variations contributes to the heterogeneity of acute myeloid leukemia (AML), a cluster of hematologic malignancies that necessitates enhanced risk evaluation for prognostic prediction and therapeutic guidance. The ubiquitin–proteasome system plays a crucial role in AML; however, the specific contributions of 49 core proteasome family members (PSMs) in this context remain largely unexplored. **Methods:** The expression and survival significance of 49 PSMs in AML were evaluated using the data from BeatAML2.0, TCGA, and the GEO database, mainly through the K-M plots, differential genes enrichment analysis, and candidate compounds screening via R language and statistical software. **Results:** we employed LASSO and Cox regression analyses and developed a model comprising three PSMs (*PSMB8*, *PSMG1*, and *PSMG4*) aimed at predicting OS in adult AML patients, utilizing expression profiles from the BeatAML2.0 training datasets. Patients with higher risk scores were predominantly found in the AML–M2 subtype, exhibited poorer ELN stratification, showed no complete remission following induction therapies, and had a higher mortality status. Consistently, significantly worse OS was observed in high-risk patients across both the training and three validation datasets, underscoring the robust predictive capability of the three-PSMs model for AML outcomes. This model elucidated the distinct genetic abnormalities landscape between high- and low-risk groups and enhanced the ELN risk stratification system. Ultimately, the three-PSMs risk score captured AML-specific gene expression signatures, providing a molecular basis for selecting potential therapeutic agents. **Conclusions:** In summary, these findings manifested the significant potential of the PSM model for predicting AML survival and informed treatment strategies.

## 1. Introduction

Acute myeloid leukemia (AML) is an aggressive and lethal disorder predominantly observed in adults, characterized by a range of subtypes that arise from the undifferentiated and hyperproliferative clonal expansion of malignant cells within the hematopoietic stem cell compartment. This disease is marked by complex karyotypes and various molecular alterations [1,2], contributing to the underappreciated heterogeneity among AML cohorts. Such genetic abnormalities lead to significantly different responses and clinical outcomes to traditional chemotherapy regimens, such as the “3 + 7” therapeutic strategy [3]. Although the management of AML patients has improved through the European LeukemiaNet (ELN) 2017 classification based on gene mutations and chromosomal aberrations [4], there remains a pressing need to develop more promising treatment options and enhanced risk stratification methodologies. The recent update to the ELN risk evaluation system in ELN2022 emphasizes the importance of genetic abnormalities [5]; however, numerous clinical trials and real-world data have indicated that this updated stratification does not substantially outperform the ELN2017 criteria in distinguishing patients’ prognostic risks [6,7,8,9]. This shortcoming underscores the necessity for more robust predictive models for AML. Notably, a considerable proportion of AML patients lack identifiable genetic alterations, making it challenging to classify their risk accurately using the existing ELN framework [2]. These observations have revealed the inherent limitations of risk models based solely on gene mutations and karyotypic abnormalities. Moreover, despite the widespread high-throughput sequencing technologies leading to the development of various predictive risk scores based on gene expression [10], alternative splicing [11], and methylation [12], consensus across multiple studies remains elusive. Consequently, there is an urgent need to identify AML-specific gene signatures that can enhance risk estimation and deepen our understanding of the disease.

The 26S proteasome, a critical component of the ubiquitin–proteasome system (UPS), comprises 19S regulatory subunits and a 20S core proteasome complex [13]. This structure is primarily responsible for the degradation of approximately 80% of misfolded and aging proteins in eukaryotic cells [14]. The 26S proteasome consists of a total of 49 proteasome family members (PSM), categorized into four classes (I–IV). This includes 19 subunits for the 20S α (*PSMA1*–*8*) and β structures (*PSMB1*–*11*), 20 subunits for 26S proteasome (ATPases for the 19S proteasome base, *PSMC1*–*6*, and *PSMCIP3*; non–ATPases for 19S proteasome lid, *PSMD1*–*14*) [15,16], as well as 4 and 1 for the proteasome activator (*PSME1*–*4*) and inhibitor (*PSMF1*), respectively, and 4 for the proteasome assembly chaperone (*PSMG1*–*4*) [17]. Among these, the constitutive proteasome subunits (CPs), which include β1 (*PSMB6*), β2 (*PSMB7*), and β5 (*PSMB5*), can be converted into distinct immunoproteasome subunits (IPs) β1i (*PSMB9*), β2i (*PSMB10*), and β5i (*PSMB8*) under interferon-gamma (IFN-γ) stimulation in hematopoietic cells and lymphoid tissue [18]. Emerging evidence has indicated that the UPS, especially the 26S proteasome, is essential for maintaining various cellular processes, including cell cycle control, DNA repair and transcription, signal transduction, and cell survival, all of which are closely linked to the initiation and development of cancer [13]. Notably, increased levels and enzymatic activity of the proteasome, along with an altered ratio of IPs to CPs, have been observed in malignant cells, leading to the development of 26S proteasome inhibitors that significantly enhance clinical response and survival outcomes in hematological malignancies, including AML [19,20].

In patients with AML, elevated proteasome levels and enzymatic activities were associated with poor clinical responses and overall survival (OS) [21,22]. Furthermore, the ratios of IPs to CPs subunits, along with aberrant expression and mutation in *PSMB5* and *PSMB8*, demonstrated significant correlations with resistance to proteasome inhibitors [23,24,25,26,27]. Additionally, increased expression of several PSMs was strongly linked to the progression from chronic myelogenous leukemia (CML) (*PSMA6*, *PSMB4*, *PSMD1/3*) [28,29], Fanconi anemia (FA) (*PSME1*) [30], and myelodysplastic syndromes (MDS) (*PSMB5*) to AML [31], as well as to poor clinical outcomes (*PSMD3*, *PSMD2*, *PSMC1*–*6*, *PSMB10*, *PSMB8*, *PSMA7*, *PSMD4*) in AML [15,32,33,34,35,36,37,38,39,40]. In contrast, the expression of *PSMD1* was deregulated during the advanced stages of MDS [41]. Notably, lower and higher expression of *PSMA2* and *PSMA6* corresponded with poor prognosis in AML [42] and were linked to higher rates of complete remission (CR) achieved in the M5 subtype [43]. Mechanistically, PSMs have been shown to promote differentiation (*PSMD4*) [44] and proliferation (*PSMB8*) [36] of leukemia cell lines through the nuclear factor kappa–B (NF–κB) and the phosphatidylinositol 3–kinase (PI3K)/protein kinase B (AKT) pathways. Conversely, the depletion of certain PSMs ex vivo enhanced apoptosis and inhibited proliferation (*PSMD1/3*, *PSME1*) [30,45,46], while deficiency in *PSMB8* impaired colony formation and delayed AML development in vivo model, extending survival [47]. These findings suggested that the 26S proteasome played a crucial role in various aspects of AML; however, the specific significance of the 49 PSMs and the central roles of individual PSMs in AML remained not fully understood.

In this study, we systematically investigated the expression and survival significance of 49 PSMs using gene expression profile, whole exon sequencing (WES) and targeted-sequencing-based mutation data, and ex vivo drug sensitivity data from BeatAML2.0, the cancer genome atlas (TCGA), and the gene expression omnibus (GEO) database. Through this comprehensive analysis, we developed a PSMs-based model to identify the most critical PSMs for AML. Our findings aimed to enhance the ELN risk classifications (both 2017 and 2022 criteria) and inform appropriate therapeutic strategies for AML patients.

## 2. Materials and Methods

### 2.1. Patients and Biological Data Resources

The patients and associated biological data utilized in this study were sourced entirely from public databases. The BeatAML2.0 dataset, which includes clinical information, normalized gene expression profile, and annotated somatic mutation data from WES and targeted sequencing data, along with ex vivo drug sensitivity data, was obtained from the research conducted by Bottomly D. et al. (https://biodev.github.io/BeatAML2/ (accessed on 16 October 2023)) [48]. This dataset served as the training set for our analysis. One of the validation sets comprised the TCGA cohorts (*N* = 179) [49], from which clinical information, normalized gene expression, and mutation annotation format (MAF) data were retrieved (https://portal.gdc.cancer.gov/ (accessed on 16 October 2023)). Additionally, we extracted gene expression profiles and survival information for the datasets GSE12417 (*N* = 78) [50] and GSE37642 (*N* = 136) [51] from the GEO, selecting these as further validation datasets.

The AML patients included in our analyses were treatment-naive, at least 18 years old, and possessed complete survival information. We assessed each patient’s risk according to the most recent ELN2022 classification within the BeatAML2.0 and TCGA datasets (Supplementary Figure S1A,B). Consistent with findings from the Beat AML cohort reported by Lachowiez CA et al., a reclassification of disease risk under the ELN2022 scheme was observed in 25% (*N* = 102) of the AML patients initially evaluated using the ELN 2017 criteria. Specifically, this included 145 cases categorized as favorable, 88 as intermediate, and 166 as adverse subgroups. In the TCGA cohort, 53, 48, and 68 cases were classified into ELN2022 favorable, intermediate, and adverse subgroups, respectively, revealing a 48% frequency (86 of 179) of risk reclassification from the ELN2017 classification, which included 37, 61, and 62 patients with the corresponding favorable (including NC (APL, acute promyelocytic leukemia)), intermediate, and adverse risks.

### 2.2. Development of PSMs-Based Prognostic Signature

The normalized TPM gene expression matrix for AML from the BeatAML2.0 dataset was utilized as the training cohort. We employed the least absolute shrinkage and selection operator (*Lasso*) analysis [52] using the Glmnet R package to identify candidate PSMs potentially associated with OS in AML. The selected genes were then incorporated into the univariate and multivariate Cox models to pinpoint the most significant prognostic PSMs related to OS. Ultimately, the PSMs identified as having independent prognostic significance through multivariate Cox analysis were used to establish a risk model, with the resulting calculation formula presented alongside a fixed intercept and coefficients (*β*) for each enrolled gene’s expression.

### 2.3. Evaluations of the Prediction Capability of the Three-PSMs Model

The predictive capability of the three-PSMs model was evaluated using both training and validation datasets. We conducted a comprehensive assessment of prediction reliability for OS through diagnostic analyses, including overall and single PSM-based, as well as time-dependent receiver operating characteristic (ROC) analysis [53] utilizing the R package pROC. Additionally, a nomograph analysis based on the multivariate Cox regression model was performed to determine whether the risk score was an independent predictor and to estimate survival probability at various time points. The nomograph calibration graph was utilized to assess the consistency between predicted and observed survival proportions of AML. Furthermore, decision curve analysis (DCA) was conducted to evaluate the potential clinical benefits of the three-PSMs model in prognosticating AML. Finally, the Harrell concordance index (C-index) derived from Cox analysis was employed to demonstrate the predictive accuracy of the three-PSMs model for OS prognosis in AML.

### 2.4. Genetic Abnormalities Landscape Analysis

The molecular alteration data in BeatAML2.0 encompassed common driver gene mutations, all available fusions from the clinical information file, and additional gene mutations collected from the annotated mutation profile derived from WES and targeted sequencing. We obtained molecular variation information for the TCGA cohort by downloading the MAF file of the annotated somatic mutation profile. A karyotype with at least three abnormalities was classified as complex. To analyze the differences in the frequency of genetic abnormalities between high- and low-risk groups based on the three-PSMs model, Fisher’s exact test was employed, and the results were visualized in a heatmap using the R package ComplexHeatmap. Significant molecular and cytogenetic alterations were depicted in a forest plot (R version: 4.3.2). A two-sided *p*-value less than 0.05 was considered statistically significant.

### 2.5. Differential Gene Analysis and GO and KEGG Enrichment Analysis

The normalized gene expression profile, measured in transcripts per million (TPM), was used to conduct differential expression analysis between the high- and low-risk groups based on the three-PSMs model using the limma R package. The criteria for the significantly expressed genes was set to an absolute log_2_(fold change (FC)) > 0.58 and an adjusted *p*-value < 0.05. The filtered differential expressed genes were visualized in volcano plots and subsequently analyzed for gene ontology (GO) and Kyoto Encyclopedia of Genes and Genomes (KEGG) enrichment using the clusterProfiler R package. The top 30 enrichment terms, ranked by gene counts and with adjusted *p*-values below 0.05, were presented in bubble charts and overlapped in a Venn diagram to illustrate shared and distinct pathways.

### 2.6. Ex Vivo Drug Sensitivity Screening

The BeatAML2.0 dataset includes an ex vivo drug sensitivity library comprising 166 compounds treated on fresh mononuclear cells (MNCs) isolated from 631 AML samples [48]. We extracted drug response data for 155 agents in 315 treatment-naive samples, each assigned the three-PSMs risk scores, ensuring that each compound had at least three tested samples in both high- and low-risk groups. The Wilcox test, conducted using R (version 4.3.2), analyzed the difference in area under the curve (AUC) for each compound between the high- and low-risk cohorts. A higher AUC indicated sensitivity to the drug, while a lower AUC signified resistance, providing insights into the therapeutic responsiveness of AML patients based on their risk stratification.

### 2.7. Statistical Analyses

The Mann–Whitney U-test was employed to compare the expression levels of 49 PSMs in AML blasts against those in healthy samples, as well as to assess the PSMs’ risk score across binary categorical clinical parameters. For the comparison of risk scores across multiple categorical clinical indicators, including AML subtype and ELN classifications from both ELN 2017 and 2022, an ordinary one-way ANOVA test was utilized. Additionally, Spearman rank analysis was conducted to evaluate the correlation between risk score and continuous variables. All these statistical analyses were executed using GraphPad Prism9.0 software to ensure accurate and reliable results.

The remaining statistical analyses were performed using R (version 4.2.1). The Survminer R package was utilized to assess the significant impact of the expression of 49 PSMs on OS in AML, producing minimal *p*-values for evaluation. Kaplan–Meier (K–M) plots and Cox proportional hazards analysis were employed to determine the significance of the three-PSMs model concerning OS. The survival duration was defined as the time from initial diagnosis to death. A two-sided *p*-value of less than 0.05 was considered statistically significant, reinforcing the robustness of our findings.

## 3. Results

### 3.1. The Expression of Most of the PSMs Alters in AML Comparing Normal Samples

**Figure 1:**
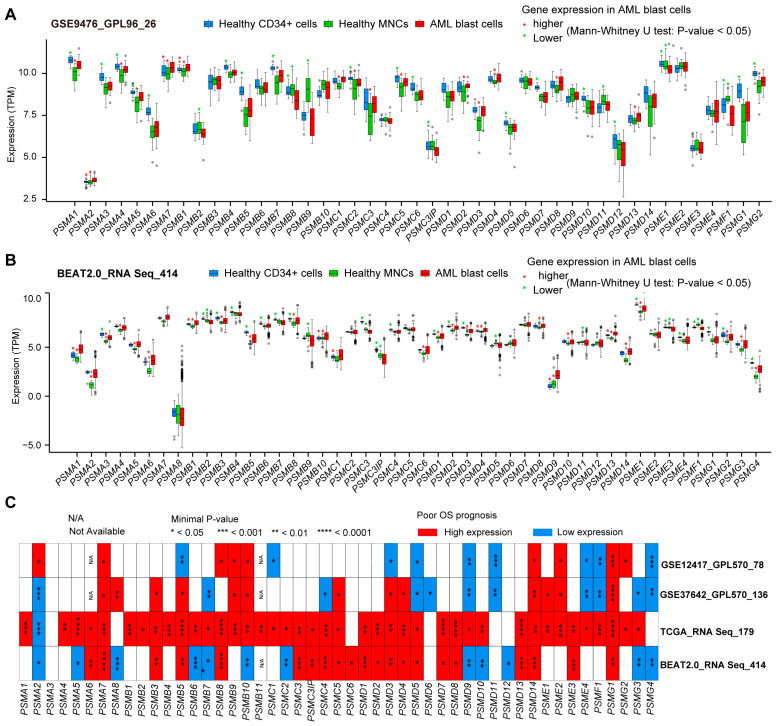
The expression of 49 PSMs and their most striking prognostic significance for OS in AML. Multiple boxplots generated using the Mann–Whitney U-test illustrated the differences in expression levels of 49 proteasome family members (PSMs) in AML blast cells compared to healthy CD34-positive and mononuclear cells from GSE9476 (**A**) and BeatAML2.0 (**B**), utilizing GPL96 and RNA-Seq as the gene detection panels, respectively. The higher and lower expressions of PSMs with *p*-values below 0.05 in AML are indicated with red and green asterisks (*) above the corresponding comparison groups. (**C**) Additionally, the panel features a heatmap displaying minimal *p*-values, highlighting the most significant associations between the expression of these 49 PSMs and OS across BeatAML2.0, the cancer genome atlas (TCGA), and two gene expression omnibus (GEO) datasets, GSE12417 and GSE37642, which employed the GPL570 expression profile panel. Statistical significance is denoted as * *p* < 0.05, ** *p* < 0.01, *** *p* < 0.001, and **** *p* < 0.0001.

To illustrate the expression patterns of the 49 PSMs in AML, we compared the expression levels of these genes in AML blast cells with those in healthy samples, which included CD34-positive cells and MNCs. This analysis utilized independent expression datasets from GSE9476 [54] (Figure 1A) and BeatAML2.0 (Figure 1B). Our findings revealed that a total of 12 PSMs exhibited higher expression in AML compared to MNCs, specifically *PSMA1*, *PSMA2*, *PSMA4*, *PSMA5*, *PSMA7*, *PSMB1*, *PSMD2*, *PSMD3*, *PSMD4*, *PSMD8*, *PSMD13*, and *PSMG2*. Notably, *PSMA1* was the only gene with significantly elevated expression in AML compared to normal CD34-positive cells. Conversely, we observed decreased expression of 10 PSMs (*PSMA3*, *PSMB4*, *PSMB5*, *PSMB7*, *PSMD1*, *PSMD12*, *PSME1*, *PSMF1*, *PSMG1*, and *PSMG2*) when comparing AML to CD34-positive cells, and 5 PSMs (*PSMB2*, *PSMB9*, *PSMC3IP*, *PSMD11*, and *PSMF1*) when compared to MNCs. Notably, *PSMF1* emerged as the only PSM with lower expression in AML than in both healthy cell types. Unfortunately, the expression levels of *PSMA8*, *PSMG3*, and *PSMG4* could not be determined due to their absence in both datasets. Overall, these results indicate that most of the 49 PSMs underwent significant expression alterations during the development of AML.

### 3.2. The Expression of PSMs Mostly Shows Close Associations with the OS of AML

Given that the majority of 49 PSMs in AML exhibited differential expression compared to normal tissues, we sought to determine whether their expression had unknown implications for AML outcomes. Survival analyses were conducted across four datasets: BeatAML2.0, TCGA, GSE12417, and GSE37642, using the expression of 49 PSMs with the minimal *p*-value as the cut-off point. As illustrated in the heatmap (Figure 1C), high expression of a total of nine PSMs (*PSMA7*, *PSMB3*, *PSMB8*, *PSMB9*, *PSMC5*, *PSMD4*, *PSMD14*, *PSME2*, and *PSMG1*) were strongly associated with adverse OS in AML, consistently observed in at least three datasets without contradictory statistical significance. Additionally, we noted that low expression of *PSMG4* was also closely linked to poor OS. These findings highlighted the potential influence of PSM expression on OS prognosis in AML, warranting further investigation.

### 3.3. Lasso and Cox Analyses Identify PSMB8, PSMG1, and PSMG4 for the OS Prognostic Model

**Figure 2:**
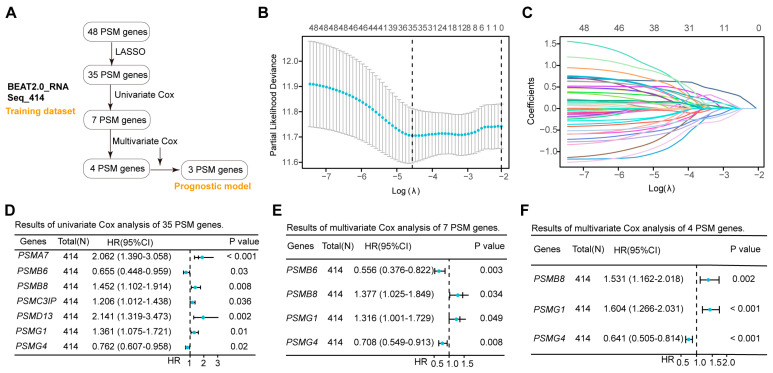
Establishment of the three-PSMs model consisting of *PSMB8*, *PSMG1*, and *PSMG4* in the training dataset. (**A**) The schematic flow chart provided an overview of the procedures used to establish the prognostic model, which included robust genes identified from 48 PSMs in the BeatAML2.0 training dataset. The lambda selection (**B**) and coefficient path (**C**) diagrams generated by the least absolute shrinkage and selection operator (*Lasso*) analysis identified 35 out of 48 PSMs with nonzero regression coefficients for further selection. Forest plots illustrate the 7 (**D**), 4 (**E**), and 3 (**F**) genes with *p*-values less than 0.05 from the sets of 35, 7, and 4, PSMs, respectively, as determined by univariate and multivariate Cox regression analyses. Ultimately, the 3 PSMs (*PSMB8*, *PSMG1*, and *PSMG4*) were identified as significant independent predictors for OS and included in the established three-PSMs model.

While the expression of most PSMs influenced the outcomes in AML positively and negatively, it remained unclear which genes played a dominant role in survival evaluations. To address this, we conducted a rigorous screening of 48 PSMs in the training dataset of BeatAML2.0, which contained the largest collection of clinical samples and associated information (Figure 2A). Initial Lasso analysis identified 35 of 48 PSMs with nonzero regression coefficients (Figure 2B,C). Given the substantial number of selected PSMs, we performed univariate Cox regression analysis and identified 7 out of the 35 PSMs that exhibited significant associations with OS in AML (Figure 2D). To enhance the robustness of the risk score based on the selected genes, we conducted a multivariate Cox regression analysis, which revealed that four of seven PSMs were independent factors (Figure 2E). However, the four-PSMs model indicated that only the expression of *PSMB8*, *PSMG1*, and *PSMG4* had significant correlations with the OS (Figure 2F). Using these three selected PSMs, we established the OS model for AML as follows: (−4.89609189549345) + *PSMB8* × 0.42609122411197 + *PSMG1* × 0.472224045804605 + *PSMG4* × (−0.444355125699367). The positive coefficients for *PSMB8* and *PSMG1*, along with the negative coefficients for *PSMG4*, indicated that high expression of *PSMB8* and *PSMG1*, as well as low expressions of *PSMG4*, were significantly associated with poor OS prognosis in AML. These findings corroborated the observations in Figure 1C., demonstrating that *PSMB8*, *PSMG1*, and *PSMG4* were likely the key PSMs linked to OS in AML and were robust for establishing a risk model.

### 3.4. The Three-PSMs OS Model Substantially Classifies the Training AML into High- and Low-Risk Groups

**Figure 3:**
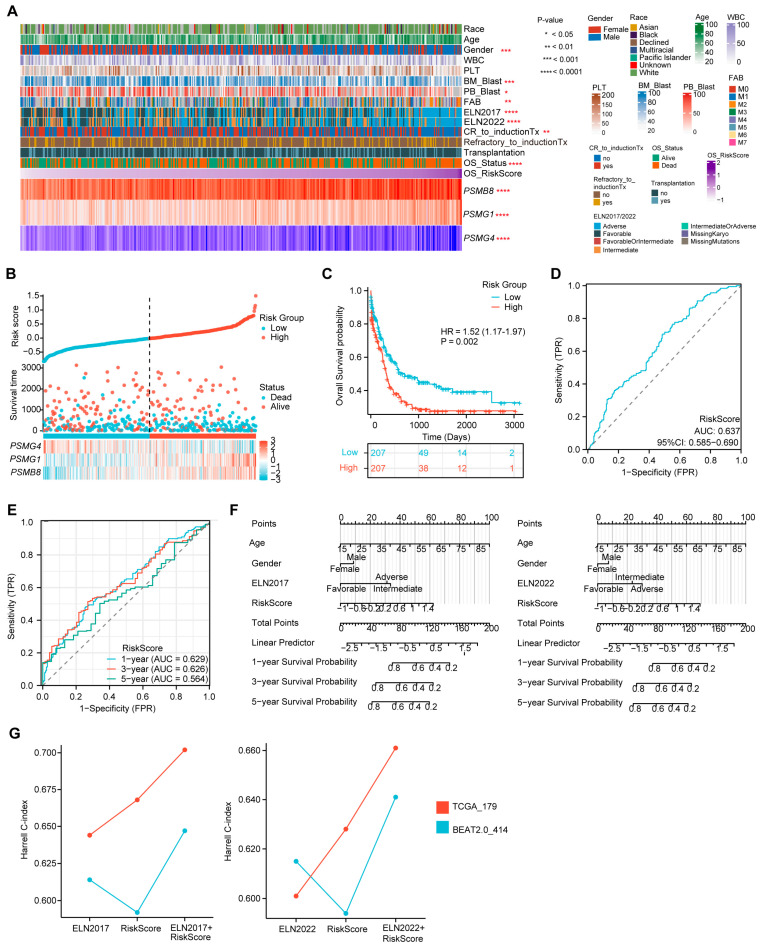
Evaluation of the prediction capability of the three-PSMs model on the OS of the training BeatAML2.0 dataset. (**A**) The heatmap illustrates the distribution of gene expression for the three-PSMs, common clinical parameters, chemotherapy responses, and clinical outcomes, highlighting their potential associations with the risk score for OS as analyzed by Spearman rank, Mann–Whitney U, and ordinary one-way ANOVA tests. (**B**) Risk factor plots categorized patients into high- and low-risk groups based on their scores and OS status. (**C**) The Kaplan–Meier (K–M) plot estimated the OS for patients in high- and low-risk categories. (**D**) The area under the curve (AUC) from diagnostic receiver operating characteristic (ROC) analysis assessed the capability of the three-PSMs score in predicting OS prognosis between high- and low-risk cohorts. (**E**) The 1-year, 3-year, and 5-year AUCs from the time-dependent ROC test demonstrated the predictive value of the 3-PSMs model for OS in patients across high- and low-risk groups. (**F**) Nomograph analysis using a multivariate Cox regression model illustrated the significance of the risk score for OS prediction, in addition to frequently assessed clinical indicators. (**G**) The Harrell C-index for the ELN classifications, the three-PSMs model, and combined cohorts were derived from decision curve analysis (DCA) involving patients from BeatAML2.0 and TCGA.

Using the established formula of the three-PSMs OS model, we calculated the risk score for each AML patient in the training dataset, reflecting the impact of proteasome genes on AML prognosis. We then analyzed the potential correlations between risk scores, clinical characteristics, and the expression of the three PSMs included in the model (Figure 3A). As anticipated, patients with high-risk scores exhibited elevated expression of *PSMB8* and *PSMG1,* along with reduced expression of *PSMG4* (Supplementary Figure S2A–C). Additionally, high-risk scores were frequently associated with a lower proportion of blast cells in both bone marrow and peripheral blood, a male predominance, and higher-risk classifications according to the ELN 2017 and 2022 guidelines, as well as the FAB (French–American–British) classification system [55], particularly in AML–M2 subtype cases (Supplementary Figure S2D–H). Notably, high-scoring patients were more likely to experience incomplete remission following induction therapies and had higher mortality rates (Supplementary Figure S2I,J), suggesting that elevated scores from the three-PSMs model corresponded to poorer OS outcomes of AML. This hypothesis was further supported by plots showing the risk score and OS status for each AML patient, with defined cut-off points for high- and low-risk groups (Figure 3B). The confirmed K–M plot indicated that patients in the high-risk group had significantly worse OS compared to those in the low-risk group (HR = 1.52, *p* = 0.002) (Figure 3C), demonstrating the model’s substantial ability to categorize AML outcomes effectively.

Furthermore, the diagnostic ROC analysis revealed an overall AUC of 0.637 (Figure 3D). The single gene-based AUCs for *PSMB8*, *PSMG1*, and *PSMG4* were 0.619, 0.570, and 0.483, respectively (Supplementary Figure S3A). In line with these results, the 1-year, 3-year, and 5-year AUCs from the time-dependent ROC curve were 0.629, 0.626, and 0.564, respectively (Figure 3E), indicating the reliability of the three-PSMs model for predicting survival probability in adult AML patients. Additionally, a nomogram analysis that incorporated age, gender, ELN 2017 and 2022 stratification, and risk score was utilized to predict the 1-year, 3-year, and 5-year cumulative survival rates of AML patients (Figure 3F). The generated calibration graph demonstrated a reasonable consistency between predicted and observed survival probabilities (Supplementary Figure S3B). DCA indicated a relatively comparable and improved predictive benefit from the combination of the risk score and ELN 2017 and 2022 classifications compared to the corresponding ELN stratification alone (Supplementary Figure S3C). The highest Harrell C-indexes from Cox regression analysis were observed in both combined groups (Figure 3G). Notably, the performance of ELN2022 in these two indexes was slightly inferior to that of ELN2017, which aligned with previous reports. Overall, these findings suggested that the risk score might enhance the ELN 2017 and 2022 classifications in assessing OS in AML. Thus, the established three-PSMs model demonstrated significant potential for classifying AML outcomes and improving the ELN risk systems.

### 3.5. The Three-PSMs Risk Score Efficiently Predicts the Survival of AML in the Validation Datasets

**Figure 4:**
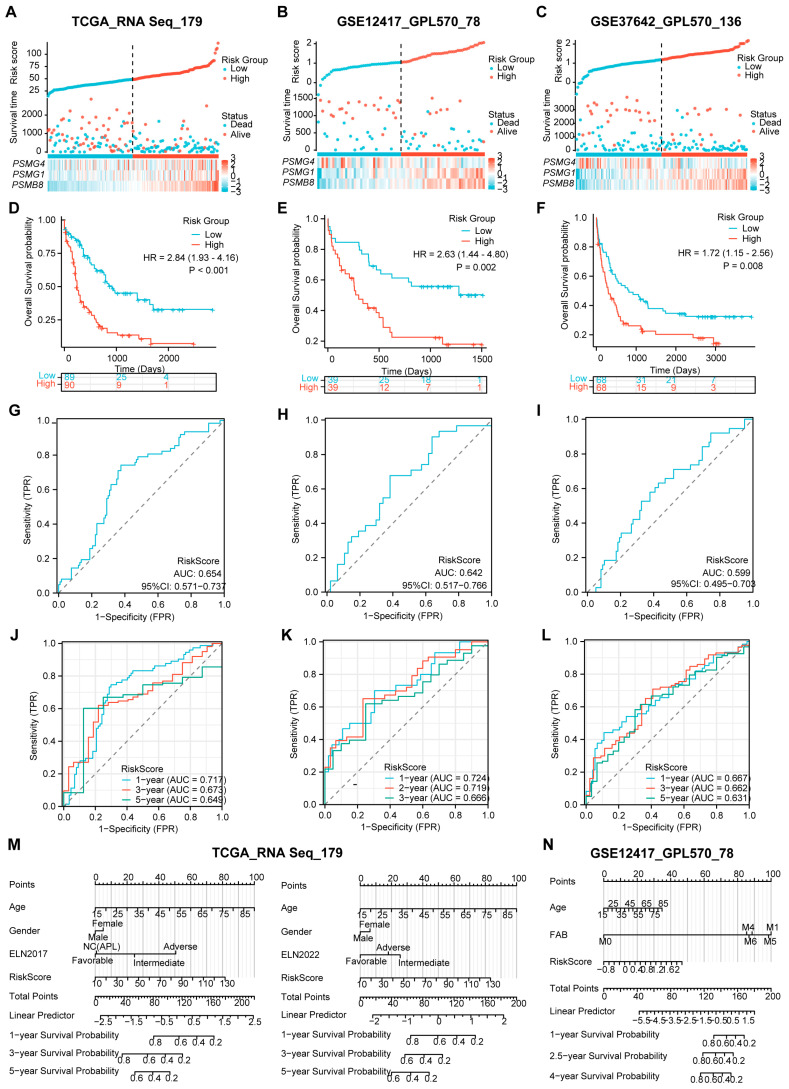
Validation of the predictive value of the three-PSMs model on OS of the independent datasets. The risk factor plots illustrate the risk score, OS status, and expression levels of the corresponding enrolled genes for each AML patient in the validation datasets from TCGA (**A**), GSE12417 (**B**), and GSE37642 (**C**), including the cut-off points used for classifying high- and low-risk groups. (**D**–**F**) The K–M plots demonstrate significant differences in OS between the high- and low-risk arms across these three gene sets. (**G**–**I**) The overall AUCs from the ROC analysis assessed the efficacy of the three-PSMs model in predicting OS for patients in high- and low-risk cohorts. Additionally, the time-dependent ROC curves (**J**–**L**) calculated the 1-year, 3-year, and 5-year AUCs for TCGA and GSE37642, as well as 1-year, 3-year, and 5-year AUCs for GSE12417, providing insights into the prediction accuracy of the risk score for OS among high- and low-risk patients. Finally, the nomograph analysis (**M**,**N**) evaluated the prognostic significance of common clinical indicators alongside the three-PSMs risk score within a multivariate Cox regression model for both TCGA and GSE12417, further underscoring the model’s utility in survival prediction.

Next, we investigated whether the risk model could effectively evaluate the survival proportions of patients across the validation datasets, which included 179 patients from TCGA, 78 patients from GSE12417, and 136 patients from GSE37642. To ensure consistency, we utilized RNA-Seq and independent GPL570 panels similar to those used in the training dataset, selecting TCGA as the internal validation and the two GEO gene sets as external validation. Using the established calculation formula, we computed the risk score for each AML case and categorized them into high- and low-risk arms based on their survival statuses (Figure 4A–C). As anticipated, patients with high-risk scores exhibited elevated expression levels of *PSMB8* and *PSMG1*, while low expression of *PSMG4* was closely associated with high-risk scores across all three validation datasets. Moreover, both internal and external gene sets validated significantly reduced OS probabilities in the high-risk group compared to those in the low-risk arm, as evidenced by the K–M plots (Figure 4D–F). These findings underscored the predictive value of the risk model concerning AML.

In addition to survival analysis, we assessed the overall AUCs via ROC analysis, which yielded values of 0.654, 0.642, and 0.599 for TCGA and the two GEO gene sets (Figure 4G–I). These results were consistent with the individual gene-based AUCs presented in Supplementary Figure S3D–F. The robustness of the prognostic risk score for OS prediction in AML was further confirmed through time-dependent ROC analysis. In this analysis, the 1-year, 3-year, and 5-year AUCs for TCGA and GSE37642 and 1-year, 2-year, and 3-year AUCs for GSE12417 ranged from 0.717, 0.667, and 0.724 to 0.649, 0.631, and 0.666, respectively (Figure 4J–L). Collectively, these data highlight the strong predictive capabilities of the risk score across the validation datasets.

Furthermore, we conducted a nomogram analysis incorporating the risk score alongside clinical factors such as patient age, gender, and ELN classifications for TCGA while using age and FAB subtype for GSE12417 (Figure 4M,N). The calibration graphs generated from the TCGA dataset, using ELN2017 rather than the 2022 version, and those from the GSE12417 demonstrated a significant agreement between the observed and predictive survival proportions (Supplementary Figure S3G,H). This finding implied a high degree of accuracy in our model for predicting AML survival outcomes. Notably, the DCA for TCGA indicated that the combination of the risk score with ELN 2017 and 2022 provided superior predictive benefits compared to either assessment alone (Supplementary Figure S3I). Cox regression analysis corroborated that this combination produced a significantly enhanced Harrell C-index compared to the separate model and the ELN 2017 and 2022 classifications (Figure 3G). Additionally, we observed the advantages of using ELN2017 alone and its combination with the three-PSMs model in both DCA and Harrer C-index analyses over those corresponding data from the ELN2022 background. These TCGA-based findings suggest that the risk score could enhance the evaluative capabilities of ELN 2017 and 2022. Overall, the results demonstrate that the three-PSMs model provided substantial prognostic insights into AML and might improve the ELN classifications within the validated datasets.

### 3.6. The Three-PSMs Risk Score Is Able to Capture the Specific Molecular and Cytogenetic Variations of AML

**Figure 5:**
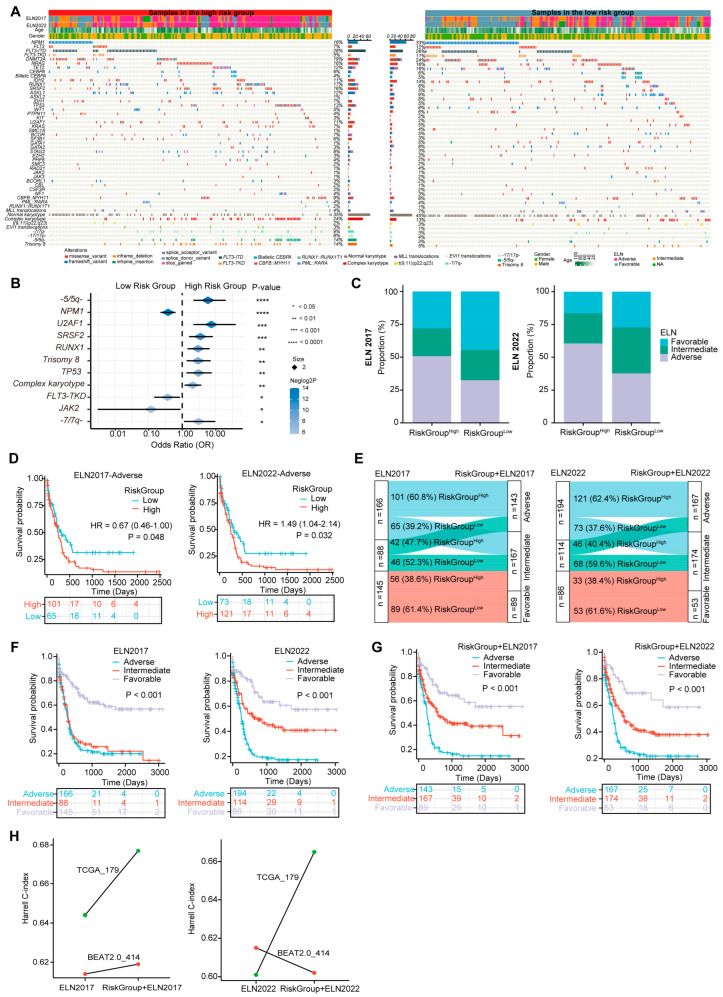
The three-PSMs model-based differential molecular and alterations and ELN reclassification in BeatAML2.0. (**A**) The heatmap generated from the chi-square test illustrates the differences in the frequencies of common gene mutations, karyotype variations, and clinical parameters between the high- and low-risk groups defined by the three-PSMs model. (**B**) The forest plot displays genetic abnormalities with differential incidences among patients in the high- and low-risk arms. (**C**) The histogram depicts the variations in proportions of European LeukemiaNet (ELN) 2017 and 2022 subgroups between the high- and low-risk cohorts. (**D**) The K–M curve estimates the OS of patients across high- and low-risk groups within the ELN 2017 and 2022 subgroups. (**E**) The Sankey chart illustrates the process of integrating the ELN 2017 and 2022 classifications with the three-PSMs score, showing how redefined favorable, intermediate, and adverse groups correspond to the patients in the low-risk category with ELN-favorable status, high-risk cohorts in the ELN-favorable group, low-risk patients in the ELN-intermediate and adverse groups, and high-risk patients classified as ELN-adverse. The K–M analysis further explored OS differences among patients with ELN 2017 and 2022 (**F**) and the refined ELN classifications using the three-PSMs model (**G**). Lastly, (**H**) the linear graph presents the Harrell C-index for risk stratification using ELN 2017 and 2022, as well as the combination of the corresponding ELN system and the three-PSMs model in the BeatAML2.0 and TCGA cohorts.

Gene mutations and karyotype abnormalities are critical marks of AML and often provide independent prognostic value for survival prediction [49]. Specific genetic alterations have been incorporated into the ELN 2017 and 2022 stratification system [4]. To comprehensively depict the genetic mutational landscape associated with the three-PSMs model, we examined common driver gene mutations and chromosome aberrations in the training and validation dataset of BeatAML2.0 (Figure 5A) and TCGA (Supplementary Figure S4). Notably, seven molecular variations and five cytogenetic abnormalities recurred with significantly different frequencies between the score-based high- and low-risk groups in the BeatAML2.0 dataset (Figure 5B). The frequencies of mutations in *U2AF1*, *SRSF2*, *RUNX1*, and *TP53,* along with chromosomal abnormalities such as *－5/5q－*, trisomy 8, complex karyotype, and *－7/7q－,* were considerably higher in the high-risk group. In contrast, mutations in the *NPM1*, *FLT3*–*TKD*, and *JAK2* were more frequent in the low-risk cohorts. K–M plots revealed that 8 of the 12 aberrant factors with higher frequencies in the high-risk group were significantly associated with poor OS (Supplementary Figure S5), corroborating findings from previous studies [5]. Consistent with the results from BeatAML2.0, the *－7/7q－* abnormality was also identified as a strong predictor of inferior prognosis among high-risk patients in the TCGA dataset (Supplementary Figure S6A,B). While the burden of other aforementioned genetic variations in TCGA did not show significant differences across the three-PSMs subgroups, a positive statistical trend emerged, indicating that specific adverse and favorable gene mutations and karyotype abnormalities occurred more frequently in the corresponding high- and low-risk patients. This finding highlighted the universal associations of established prognostic factors with our risk model (Supplementary Table S1). These observations were further substantiated by the higher proportions of genetic lesions corresponding to favorable and adverse ELN classifications in the high- and low-risk three-PSMs arms, irrespective of the ELN version applied. The *PML::RARA* fusion was another notable alteration, which exhibited a significantly higher frequency and a relatively better OS in the low-risk TCGA cohorts. However, multivariate Cox regression analysis, incorporating all significantly differential molecular and cytogenetic alterations, common clinical indicators, and the three-PSMs score, indicated that only *NPM1* and *TP53* retained prognostic significance in BeatAML2.0. In contrast, the three-PSMs model consistently functioned as an independent prognostic factor in both the BeatAML2.0 and TCGA datasets, regardless of the ELN scheme employed (Supplementary Tables S2 and S3). These data suggest that the three-PSMs model could effectively distill the differential landscape of established genomic abnormalities with their corresponding well-known prognostic implications.

### 3.7. The Three-PSMs Model Can Compensate for the ELN Classification

Given that the driver molecular and cytogenetic variations critically defining the ELN scheme [4] could be captured by the three-PSMs model, we next explored the predictive value of this risk score within the contexts of both the ELN2017 and ELN2022 criteria. The nomogram analyses conducted through multivariate Cox regression tests indicated that the risk model served as an independent prognostic factor alongside patient age and the ELN classifications in the BeatAML2.0 and TCGA datasets (Figure 3F and Figure 4M). Additionally, the significant differences or strong statistical trends observed in the proportions of genetic alterations between the three-PSMs subgroups further validated the robustness of our risk model (Figure 5B and Supplementary Figure S6A and Table S1). Interestingly, the distribution of ELN stratifications was notably imbalanced between the three-PSMs’ high- and low-risk cohorts, particularly within the ELN2017 framework in the BeatAML2.0 dataset (Figure 5C, Supplementary Figure S6C). Specifically, nearly 50% of patients in the high-risk group were classified as adverse, while almost the same proportion in the low-risk group fell into the favorable classifications of ELN2017. However, 28.1% and 21.1% of cases in the high-risk arm were classified as favorable and intermediate groups, respectively, while 23.0% and 32.5% of low-risk patients were assigned to intermediate and adverse categories of ELN2017. Furthermore, the prognostic model effectively distinguished significant differences in OS among subgroups with intermediate and adverse ELN risks, regardless of the ELN criterion applied (Figure 5D, Supplementary Figure S6D). In addition to insights from DCAs, which demonstrated a higher concordance for survival prediction when combining the current model with both ELN systems (Figure 3G), we sought to redefine the disease risk of patients by incorporating the three-PSMs model into the ELN 2017 and 2022 frameworks (Figure 5E, Supplementary Figure S6E). According to this new categorization, patients in the low-risk group with ELN-favorable status remained in the favorable-risk group, while high-risk cohorts with ELN-favorable status were reassigned, and low-risk patients in the ELN-intermediate and adverse groups were moved to the intermediate-risk arm. Patients with high risk and ELN-adverse status were reclassified as the adverse-risk group. Notably, given their similar OS, the ELN2017–NC (APL) cases in TCGA were treated as ELN2017-favorable. As anticipated, both redefined risk schemes resulted in significant improvements in the prognostic stratification of AML patients, as illustrated in the K–M plots, particularly for the combined ELN2017 classifications across both cohorts (Figure 5F,G, Supplementary Figure S6F,G). Consistently, the higher Harrell C-index in the recategorized BeatAML2.0 and TCGA gene sets further supported the superiority of the renewed ELN2017 classification (Figure 5H). In contrast, the ELN2022 system yielded a decreased index when combined with the three-PSMs model, emphasizing the compatibility of ELN2017 with our risk score. We theorized that this suboptimal outcome might align with the previously noted slight inferiority of ELN2022 compared to ELN2017 alone (Figure 3G, Supplementary Figure S3C). These findings collectively demonstrate that the three-PSMs model possessed significant prognostic value in enhancing the ELN classifications.

### 3.8. The Three-PSMs Score Can Identify AML-Specific Gene Expression Signatures and Provide Suitable Therapeutic Guides for Patients

**Figure 6:**
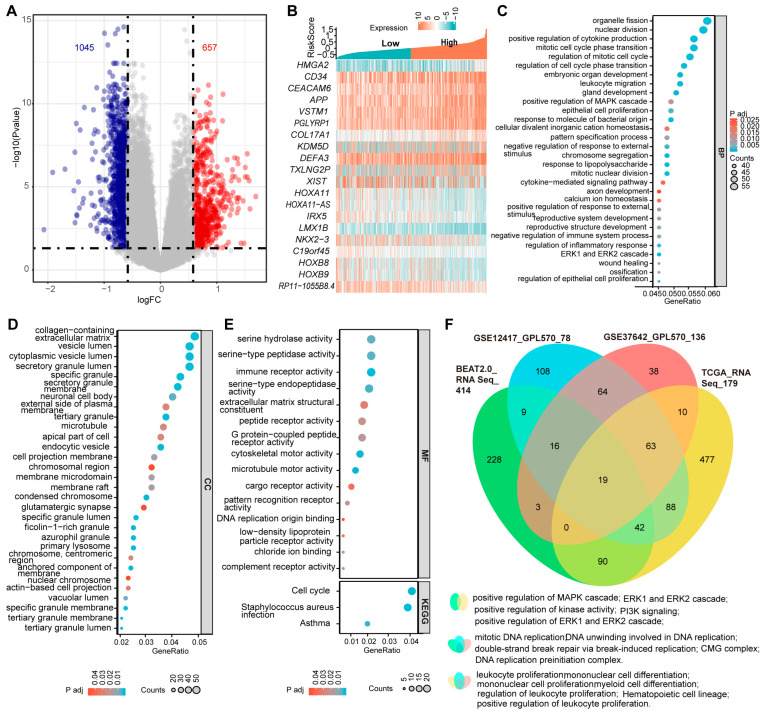
Differentially expressed genes and the enrichment data between the three-PSMs high- and low-risk groups in BeatAML2.0. (**A**) The volcano plot illustrates the significantly differentially expressed genes with an absolute log_2_(FC) > 0.58 between the high- and low-risk arms. (**B**) The heatmap displays the top 10 upregulated and downregulated genes among patients with high- and low-risk groups. (**C**–**E**) The bubble charts present the top 30 enrichment results from gene ontology (GO) and the Kyoto Encyclopedia of Genes and Genomes (KEGG) analyses based on the differentially expressed genes in the high- and low-risk cohorts. The GO analysis encompassed biological process (BP), cellular component (CC), and molecular function (MF). (**F**) The Venn diagram illustrates the shared GO and KEGG enrichment data that were significantly associated with the initiation and development of AML across BeatAML2.0 and the three validation datasets.

The complexity of genetic alterations in AML leads to significant dysregulation of specific genes at the transcriptional level, resulting in a unique and heterogeneous transcriptomics profile associated with the disease [3]. To gain deeper insights into the molecular basis of AML, we conducted differential gene expression analyses between samples with the three-PSMs high- and low-risk groups in the training BeatAML2.0 and three validation datasets. In BeatAML2.0, we identified a total of 1045 deregulated and 657 upregulated genes, with an absolute Log_2_(FC) > 0.58 and an adjusted *p*-value < 0.05 (Figure 6A). The validation datasets revealed 588 and 719 differential genes in TCGA, 179 and 668 in GSE12417, and 134 and 468 in GSE37642, respectively (Supplementary Figure S7A–C). Notably, many of the top 10 differentially expressed genes were found to be associated with key processes such as stemness (*CD34* and *HLA*–*DR*) [56], leukemogenesis and progression (*CRNDE*, *HMGA2*, *KDM5D*, and *HOXB8*) [57,58,59,60], and chemotherapy resistance (*HOXA11* and *LIN7A*) [61,62] (Figure 6B, Supplementary Figure S7D–F). Other genes, such as *EPDR1* [63] and *HOXA11*–*AS* [64]*,* identified as critical factors for cancer growth and metastasis, warranted further investigation in the context of AML. Importantly, we observed that cell cycle signaling pathways, which played central roles in AML, were consistently enriched among the top 30 categories in GO enrichment analyses and corroborated by KEGG data from the BeatAML2.0 dataset (Figure 6C–E). When we cross-referenced these enrichment data with the three validation datasets, we found that the MAPK, ERK1, and ERK2 signaling cascades, identified among the top 30 in the GO analysis of BeatAML2.0, were also enriched in the TCGA (Figure 6C,F, Supplementary Figure S8). Other shared enrichment results included pathways related to DNA replication and double-strand break repair, and myeloid, mononuclear, and leukocyte cell proliferation and differentiation, as well as hematopoietic cell lineage, all of which are significantly associated with initiation, development, and biological properties of AML (Figure 6F, Supplementary Figure S8). Collectively, these findings demonstrated that the three-PSMs model could effectively identify AML-specific gene expression signatures that might contribute to leukemogenesis and chemotherapy resistance.

**Figure 7:**
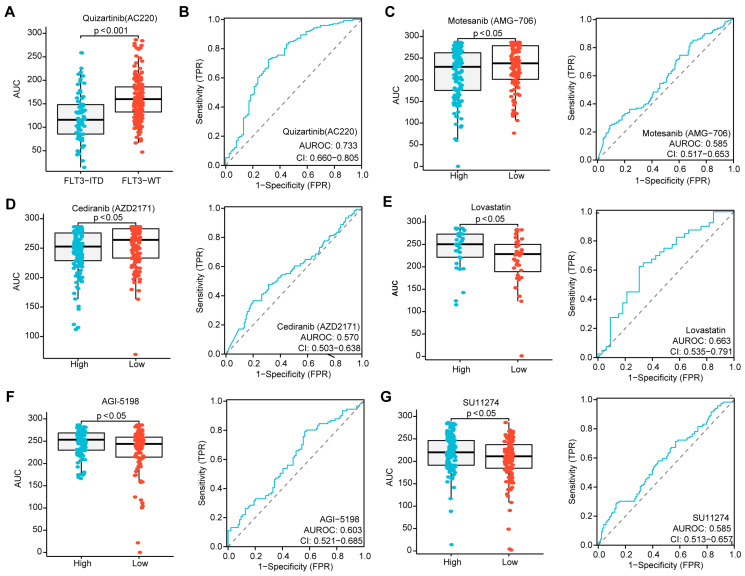
Identification of agents with significantly differential drug sensitivity by the three-PSMs model. (**A**) The boxplot generated using the Wilcox test demonstrates a significantly different drug response to Quizartinib (AC220) among patients with *FLT3*–*ITD* mutation compared to those with wild-type (WT) *FLT3*, with higher AUCs indicating sensitivity and lower AUCs indicating resistance to the compounds. (**B**) The ROC curve assesses the reliability of the differential effects of Quizartinib (AC220) on *FLT3*–*ITD*-positive versus WT–*FLT3* patients. Further, the boxplots and ROC analyses highlight the difference in drug sensitivity of Motesanib (AMG–706) (**C**), Cediranib (AZD2171) (**D**), Lovastatin (**E**), AGI–5198 (**F**), and SU11274 (**G**) between the high- and low-risk groups defined by the three-PSMs model. The *p*-value less than 0.05 was determined as having statistical significance.

Given that the risk score in patients without CR was higher than in those who achieved CR during induction chemotherapy, and that a higher score was associated with inferior OS in AML, we sought to determine whether the three-PSMs risk score could identify candidate agents for treatment options. Utilizing the ex vivo drug sensitivity library from BeatAML2.0, we generated a drug-response AUC profile that included 166 agents tested across 631 samples. To ensure the reliability of this evaluation, we selected Quizartinib, a well-known second-generation FLT3 inhibitor approved by the FDA for treating relapsed or refractory AML with FLT3–ITD mutation, as the positive control [65]. As anticipated, we observed a significantly lower AUC for Quizartinib, indicating sensitivity in patients with FLT3–ITD mutation, while those with wild-type FLT3 did not show this signal (Figure 7A). The ROC curve analysis further suggested that FLT3–ITD-positive patients exhibited higher drug sensitivity values compared to wild-type patients (Figure 7B). Next, we compared the differential AUCs of 155 compounds across 315 samples between high and low three-PSMs-risk groups using the Wilcox test. Notably, we identified two agents, Motesanib (AMG–706) and AGI–5198, that demonstrated significantly lower AUCs, as well as three agents (Lovastatin, SU11274, and Cediranib (AZD2171)) with higher AUCs and elevated ROC sensitivity values in the high-risk cohorts. These findings corresponded to potential high-risk three-PSMs sensitive (Figure 7C,D) and resistant (Figure 7E–G) compounds, respectively. Most of these compounds have been reported to be effective in treating solid tumors by targeting multiple kinases, thereby influencing downstream signalings such as MAPK–ERK, PI3K–Akt, and JAK–STAT [66]. Interestingly, these kinase pathways, including the MAPK, ERK1, and ERK2 cascade, as well as PI3K signaling, were also enriched in the GO and KEGG analyses of differential genes between high and low three-PSMs-risk cohorts (Figure 6C,F, Supplementary Figure S8). This correlation helped us to elucidate the underlying molecular mechanisms through which these compounds might exert their effects in AML. Overall, these results manifested that the three-PSMs score could serve as a predictive tool for drug sensitivity, guiding therapeutic strategies. However, further exploration was needed to specifically assess the effects of the selected agents on AML cells.

## 4. Discussion

The development of effective risk stratification for AML is both critical and urgent, as it aims to accurately assess disease severity and provide appropriate therapeutic regimens for patients. The established ELN2017 classification, along with the more recent ELN2022 update, efficiently evaluates the risks of AML patients based on karyotype detection and specific gene mutation analysis [4,5]. However, the diverse genetic abnormalities inherent to AML contribute to its complexity and heterogeneity, possibly impairing the efficacy of these risk classification systems in clinical practice [3]. Notably, a significant proportion of AML patients lack specific gene mutations and present with normal karyotypes, making the ELN risk stratification less applicable. Fortunately, the widespread and reliable usage of high-throughput approaches, particularly transcriptomic sequencing, offers a significant advantage by providing emerging prognostic information at the transcriptomic level for AML [10,11,67]. In the present study, we hypothesized that the transcriptome profile constructed from 26S proteasome-related 49 PSMs might carry important prognostic significance for OS in AML. By establishing a predictive risk model, we identified a robust prognostic signature comprising three PSMs: *PSMB8*, *PSMG1*, and *PSMG4.* This signature was validated in three independent validation gene sets using distinct gene detection panels and data analytical platforms (Supplementary Table S4). Our research demonstrated that the PSM expression signature might be independent of the genetic abnormalities commonly observed in AML, distilling it into a universal feature that predicted prognosis. As previously reported, altered expression of PSMs likely influenced pathways associated with the survival and growth of malignant cells [13,14], resulting in differential OS among AML subgroups.

The three-PSMs risk score significantly reflected the impact of genetic lesions and clinical parameters on the survival advantage of leukemia cells in AML. It is generally acknowledged that inferior prognostic markers are often associated with chaotic biological mechanisms that can confer increased aggressiveness to the malignant cells. Unsurprisingly, cohorts within the high-risk group of the three-PSMs model exhibited a significantly greater burden of multiple adverse prognostic factors in AML (Supplementary Table S4), including mutations in *TP53*, *RUNX1*, *U2AF1*, and *SRSF2*, as well as cytogenetic abnormalities such as *－5/5q－*, trisomy 8, *－7/7q－*, and complex karyotype [68]. Notably, the MDS-related mutations in *U2AF1* and *SRSF2* have been recently included in the ELN2022 classification [5], emphasizing the reliability of the three-PSMs model in distilling molecular alterations associated with AML. We posited that these adverse prognostic markers likely promoted leukemia by enhancing the competitiveness of the leukemic cells [69] and providing resistance to the hostile environment [70], ultimately leading to the expansion and accumulation of dominant subclones. This speculation was supported by the prominent enrichment of various cell survival signalings, including MAPK, ERK1/2, and PI3K, as well as functions related to myeloid, mononuclear, and leukocyte cell proliferation. Interestingly, the poor prognosis associated with the three-PSMs model also appeared to correlate significantly with the AML–M2 subtype, which typically harbors the favorable prognostic factor of *CBF* fusions. We inferred that this unexpected finding might stem from the relatively small proportion of *CBF* rearrangements observed in both the BeatAML2.0 (15%, 8/52) and TCGA (15%, 6/41) datasets. Moreover, we noted higher frequencies of favorable prognostic factors, such as *NPM1* mutation and *PML::RARA* fusion, in the three-PSMs low-risk arm; however, only the *PML::RARA* rearrangement maintained its positive impact among the three-PSMs subgroups. This observation could be attributed to the almost complete mutual exclusion of *NPM1* mutation and *PML::RARA* fusion. The favorable prognostic effects of these gene variations might be neutralized when assigned to opposite risk groups within the three-PSMs model. Nevertheless, the *NPM1* mutation, along with mutated *TP53,* remained an independent predictor among these genetic abnormalities and clinical factors [68]. Importantly, the three-PSMs score demonstrated strong independence from molecular, cytogenetic, and clinical markers, emphasizing the influence of AML-driver gene mutations on the PSMs-conferred survival properties of leukemic cells. Thus, the three-PSMs model was well equipped to capture downstream AML-related alterations and signatures, further enhancing its utility in clinical prognostication.

The strong prediction capacity of the three-PSMs score also provided additional prognostic information and compensated for the limitations of the ELN classification system (Supplementary Table S4). By integrating the three-PSMs model, the redefined ELN risk system demonstrated a more accurate classification, evidenced by improved survival prognoses among subgroups, particularly for patients initially categorized within the original ELN-intermediate and -adverse risk groups. This enhancement of the ELN classification through the three-PSMs model might facilitate a more reliable evaluation for newly diagnosed AML patients, thereby aiding in the development of more tailored therapeutic strategies. In this context, our study preliminary identified several potential agents that exhibited either sensitivity or resistance in relation to the high three-PSMs score. Interestingly, many of these compounds target the activity of tyrosine kinases and their downstream signalings, which primarily mediate the survival and proliferation of malignant cells [66]. This finding aligned with the enrichment analysis associated with the three-PSMs scores, providing valuable guidance for candidate drug profiles in clinical regimes. However, the specific effects and underlying mechanisms of these agents on leukemic cells require further investigation through ex vivo and in vivo models in future studies.

Despite the promising findings of our research, several potential limitations warranted attention for future improvement (Supplementary Table S5). One notable concern was the relatively small size and single origin of the samples in the validation cohort, as nearly all validated patients were drawn from Western public databases. This might introduce potential biases related to race and socioeconomic status that could influence clinical outcomes. To enhance the applicability of the three-PSMs model, future studies should include real-world data from diverse racial backgrounds to provide more objective and convincing insights. Additionally, our study primarily focused on relative evaluations and lacked basic experimental data. The three prognostic PSMs identified as the foundation for developing the prognostic score should benefit from validation using leukemia cell lines or fresh patient samples. It also raised much interest in investigating whether the candidate compounds could effectively inhibit the viability of leukemic cells. Another area of confusion pertained to the validity of the ELN2022 stratification; its prognostic significance showed contradictions across different parameters. Although our analyses indicated a somewhat poorer performance of ELN2022 compared to ELN2017, as evidenced by DCA and Harrell C-index results in both public cohorts, the OS in K–M plots displayed significant differences across ELN2022 subgroups in the BeatAML2.0, aligning closely with previous studies. Thus, further research on the risk estimation of ELN2022 in AML is warranted, utilizing larger-scale patient datasets from multiple origins.

The inclusion of *PSMB8*, *PSMG1*, and *PSMG4* enrolled in the three-PSMs model more likely highlighted the most significant PSMs related to OS in AML among the 26S proteasome components. *PSMB8,* along with *PSMB9* and *PSMB10*, forms part of the IPs [18], where abnormal expression, altered activity, and mutation have been shown to significantly mediate resistance to 26S proteasome inhibitors [23,24,25,27]. Previous studies suggested that the deregulation of IPs could lead to immune escape in APL cells during the progression of the disease, a process that might be reversed by the all-trans retinoic acid (ATRA) [71]. Consistent with our findings, several sporadic studies reported elevated expression of *PSMB8* in AML, likely due to the influence of the long noncoding RNA–HLA complex P5 (*HCP5*), and this elevation correlated with poor clinical outcomes [36,37,38,39]. Additionally, the upregulation of *PSMB8* in cancer appeared to occur through a cell-intrinsic mechanism, being more prevalent in the AML–M5 subtype, which frequently harbors myeloid/lymphoid or mixed-lineage leukemia (*MLL*) fusions [72]. Critically, *PSMB8* was responsible for the self-renewal capacity of leukemia stem cells and the initiation of *MLL*-rearranged leukemia by regulating the DNA binding with brain abundant membrane attached signal protein 1 (*BASP1*), positioning it as a promising target for anti-leukemia therapy [47]. Indeed, *PSMB8* played a vital role in multiple aspects of AML, further affirming the reliability of the three-PSMs model. However, the effects of *PSMG1* and *PSMG4* on AML and other hematologic malignancies remained largely unexplored, warranting further investigation. *PSMG1* and *PSMG4* are part of the proteasome assembly chaperone family of 26S proteasome [17], primarily mediating proteasome maturation. High expression levels of *PSMG4* were identified as a potential biomarker for adverse OS in lung cancer [73], while *PSMG1* showed prognostic significance at the pan-cancer level [17]. Notably, *PSMG1*, also known as Down Syndrome Critical Region Gene 2 (*DSCR2*), was implicated in Down Syndrome harboring trisomy 21 [74], which was associated with an increased risk of developing AML [75]. These findings suggest that *PSMG1* and *PSMG4* might be involved in various cancers, including AML, and highlight the need for further research to elucidate their roles in these malignancies.

## 5. Conclusions

In conclusion, through an analysis of transcriptomic profile, WES and targeted sequencing data, and ex vivo drug sensitivity data from five independent sets, we comprehensively characterized the altered expression and survival significance of 49 PSMs for the 26S proteasome in AML. This research led to the development of a substantially powerful prognostic model based on three key PSMs: *PSMB8*, *PSMG1*, and *PSMG4,* which were confirmed as independent predictors of OS in AML. The three-PSMs model effectively captured the AML-driver molecular mutations and karyotype abnormalities, along with the AML-specific differential expression gene signatures, providing valuable molecular foundations for guiding clinical treatment decisions. Furthermore, the newly redefined ELN2017 scheme, incorporating the three-PSMs score, facilitated more accurate classifications and improved survival prognostication among AML subgroups, underscoring the model’s reliability in evaluating patient risk for clinical application.

## Data Availability

The original contributions presented in the study are included in the article/supplementary material; further inquiries can be directed to the corresponding author.

## Supplementary Materials

The following supporting information can be downloaded at: https://www.mdpi.com/article/10.3390/biomedicines12092147/s1, Supplementary Table S1: The clinical, cytogenetic, and molecular characteristics of AML by the three-PSMs classification; Supplementary Table S2: Multivariate Cox analysis of the three-PSMs score, genetic abnormalities, and clinical parameters in BeatAML2.0; Supplementary Table S3: Multivariate Cox analysis of the three-PSMs score, genetic abnormalities, and clinical parameters in TCGA; Supplementary Table S4: Main findings concerning our developed three-PSMs risk system in BeatAML2.0, TCGA, and GEO datasets; Supplementary Table S5: Lists of potential limitations of our study which should be improved in the future; Supplementary Figure S1: The distribution of the ELN2022 prognostic scheme by reclassifying the 2017 version; Supplementary Figure S2: The relationship of the three-PSMs score with gene expression and clinical parameters in AML; Supplementary Figure S3: Estimation of the prediction capability of the three-PSMs model for AML; Supplementary Figure S4: The three-PSMs model-based genetic abnormalities landscape in TCGA; Supplementary Figure S5: The overall survival analysis of specific genetic alterations in the BeatAML2.0 cohorts; Supplementary Figure S6: The optimization of the three-PSMs model for ELN stratification in TCGA; Supplementary Figure S7: Differential gene expression profile between the three-PSMs high- and low-risk groups in the validation datasets; Supplementary Figure S8: The top-30 enrichment data of GO and KEGG analysis of the three-PSMs-based differential expressed genes.
