# Supplementary material for: The Proteasome-Family-Members-Based Prognostic Model Improves the Risk Classification for Adult Acute Myeloid Leukemia
Source: Biomedicines. 2024 Sep 22;12(9):2147. doi: 10.3390/biomedicines12092147 (PMC11429122; doi:10.3390/biomedicines12092147)
Supplement: Supplementary file 1 [file biomedicines-12-02147-s001.zip › biomedicines-3153212-supplementary.pdf]

Supplementary Information

Supplementary Tables

Supplementary Table S1. The clinical, cytogenetic, and molecular characteristics of AML by the three–PSMs classification.

**Parameters**	**Training BeatAML2.0 cohort**			**Validating TCGA cohort**		
	High (*N*=207)	Low (*N*=207)	*p* value	High (*N*=90)	Low (*N*=89)	*p* value
Median age at diagnosis (range)	62 (18-88)	61 (20-84)	0.3817	61 (18-88)	55 (21-82)	**0.0224**
Male:Female	128:79	98:109	**0.0041**	52:38	46:43	0.2322
WBC×10^9/L,median (range)	*N*=174 22.87 (0.5-427.46)	*N*=179 27.17 (0.9-230.05)	0.5272	26 (0.8-223.8)	14.3 (0.4-297.4)	0.0789
FAB subtype *N*, %	*N*=133	*N*=120	0.1457	*N*=88	*N*=89	**0.0008**
M0	8(6.02%)	10(8.33%)		7(7.95%)	9(10.11%)	
M1	25(18.80%)	32(26.67%)		23(26.14%)	19(21.35%)	
M2	34(25.56%)	18(15.00%)		21(23.86%)	20(22.47%)	
M3	9(6.77%)	9(7.50%)		1(1.14%)	15(16.85%)	

M4	31(23.31%)	18(15.00%)		15(17.05%)	21(23.60%)	
M5	23(17.29%)	29(24.17%)		16(18.18%)	5(5.62%)	
M6	1(0.75%)	3(2.50%)		2(2.27%)	0	
M7	2(1.50%)	1(0.83%)		3(3.41%)	0	
Risk group (2017ELN) *N*, %	*N*=199	*N*=200	**0.0042**	*N*=89	*N*=74	**0.0003**
Favorable	56(28.14%)	89(44.50%)		15(16.85%)	23(31.08%)	
Intermediate	42(21.11%)	46(23.00%)		35(39.33%)	28(37.84%)	
Adverse	101(50.75%)	65(32.50%)		39(43.82%)	23(31.08%)	
Risk group (2022ELN)*N*, %	*N*=200	*N*=194	**<0.0001**	*N*=84	*N*=85	**0.0365**
Favorable	33(16.50%)	53(27.32%)		22(26.19%)	31(36.47%)	
Intermediate	46(23.00%)	68(35.05%)		20(23.81%)	28(32.94%)	
Adverse	121(60.50%)	73(37.63%)		42(50.00%)	26(30.59%)	
Risk group (cytogenetic)*N*, %				*N*=87	*N*=89	**0.0016**
Good	NA	NA	NA	8(9.20%)	25(28.09%)	
Intermediate	NA	NA	NA	52(59.77%)	49(55.06%)	
Poor	NA	NA	NA	27(31.03%)	15(16.85%)	
Cytogenetic abnormality *N*, (%)	*N*=199	*N*=192		*N*=88	*N*=87	

Normal	70(35.18%)	86(44.79%)	0.0629	38(43.18%)	38(43.68%)	1.0000
Complex	47(23.62%)	25(13.02%)	**0.0088**	16(18.18%)	11(12.64%)	0.4032
11q23//MLL translocations	8(4.02%)	14(7.29%)	0.1906	5(5.68%)	5(5.75%)	1.0000
t(9;11)(p22;q23)	4(2.01%)	6(3.13%)	0.5371	1(1.14%)	0	1.0000
3q26/EVI1 translocations	6(3.02%)	2(1.04%)	0.2846	2(2.27%)	0	0.4971
—7/7q—	18(9.05%)	6(3.13%)	**0.0192**	14(15.91%)	4(4.60%)	**0.0229**
—17/17p-	16(8.04%)	8(4.17%)	0.1405	6(6.82%)	3(3.45%)	0.4963
—5/5q—	27(13.57%)	5(2.60%)	**< 0.0001**	10(11.36%)	5(5.75%)	0.2801
Trisomy 8	28(14.07%)	10(5.21%)	**0.0034**	8(9.09%)	10(11.49%)	0.6281
Molecular fusions *N*, %	*N*=199	*N*=192		*N*=88	*N*=87	
PML::RARA	7(3.52%)	8(4.17%)	0.7968	1(1.14%)	15(17.24%)	**0.0060**
CBFB::MYH11	17(8.54%)	16(8.33%)	1.0000	4(4.55%)	7(8.05%)	0.3706
RUNX1::RUNX1T1	3(1.51%)	2(1.04%)	1.0000	3(3.41%)	4(4.60%)	0.7201
Gene mutations *N*, %	*N*=199	*N*=192		*N*=88	*N*=87	
NPM1	31(15.58%)	63(32.81%)	**< 0.0001**	13(14.77%)	7(8.05%)	0.2345
FLT3 (other)	13(6.53%)	23(11.98%)	0.0796	14(15.91%)	6(6.90%)	0.0945
FLT3–ITD	56(28.14%)	50(26.04%)	0.6509	19(21.59%)	17(19.54%)	0.8520

FLT3–TKD	9(4.52%)	22(11.46%)	**0.0142**	7(7.95%)	4(4.60%)	0.5355
DNMT3A	37(18.59%)	46(23.96%)	0.2169	11(12.5%)	9(10.34%)	0.8129
NARS	32(16.08%)	30(15.63%)	1.0000	6(6.82%)	4(4.60%)	0.7466
TET2	24(12.06%)	31(16.15%)	0.3085	1(1.14%)	3(3.45%)	0.6207
CEBPA	11(5.53%)	11(5.73%)	1.0000	3(3.41%)	1(1.15%)	0.6207
Biallelic *CEBPA*	7(3.52%)	9(4.69%)	0.6165	4(4.55%)	1(1.15%)	0.3678
IDH2	22(11.06%)	27(14.06%)	0.4453	7(7.95%)	4(4.60%)	0.5355
RUNX1	33(16.58%)	12(6.25%)	**0.0014**	10(11.36%)	3(3.45%)	0.0804
SRSF2	32(16.08%)	10(5.21%)	**0.0005**	13(13.04%)	6(8.05%)	0.3315
ASXL1	23(11.56%)	18(9.38%)	0.5126	4(4.55%)	2(2.30%)	0.6820
ASXL2	1(0.50%)	0	1.0000	1(1.14%)	1(1.15%)	1.0000
IDH1	13(6.53%)	16(8.33%)	0.5648	2(2.27%)	4(4.60%)	0.4436
TP53	26(13.07%)	9(4.69%)	**0.0042**	8(9.01%)	5(5.75%)	0.5660
WT1	10(5.03%)	15(7.81%)	0.3042	3(3.41%)	9(10.34%)	0.0804
PTPN11	8(4.02%)	12(6.25%)	0.3638	4(4.55%)	3(3.45%)	1.0000
KIT	5(2.51%)	3(1.56%)	0.7241	4(4.55%)	5(5.75%)	0.7466
U2AF1	21(10.55%)	3(1.56%)	**0.0002**	5(5.68%)	2(2.30%)	0.4436

KRAS	14(7.04%)	9(4.69%)	0.3923	3(3.41%)	3(3.45%)	1.0000
SMC1A	2(1.10%)	7(3.65%)	0.0999	3(3.41%)	2(2.30%)	1.0000
BCOR	13(6.53%)	9(4.69%)	0.5128	3(3.41%)	1(1.15%)	0.6207
SF3B1	12(6.03%)	5(2.60%)	0.1358	1(1.14%)	2(2.30%)	0.6207
GATA1	2(1.01%)	0	0.4989	0	0	1.0000
GATA2	5(2.51%)	9(4.69%)	0.2845	2(2.27%)	3(3.45%)	0.6820
STAG2	16(8.04%)	15(7.81%)	1.0000	0	1(1.15%)	0.4971
EZH2	5(2.51%)	6(3.13%)	0.7677	3(3.41%)	1(1.15%)	0.6207
PHF6	7(3.52%)	6(3.13%)	1.0000	1(1.14%)	3(3.45%)	0.3678
SMC3	4(2.01%)	3(1.56%)	1.0000	2(2.27%)	3(3.45%)	0.6820
RAD21	2(1.10%)	3(1.56%)	0.6804	1(1.14%)	2(2.30%)	0.6207
JAK2	1(0.50%)	8(4.17%)	**0.0183**	0	0	1.0000
JAK3	2(1.01%)	1(0.52%)	1.0000	1(1.14%)	0	1.0000
BCORL1	6(3.02%)	3(1.56%)	0.5034	4(4.55%)	6(6.90%)	0.5355
CBL	4(2.01%)	3(1.56%)	1.0000	2(2.27%)	2(2.30%)	1.0000
CSF3R	3(1.51%)	1(0.52%)	0.6233	1(1.14%)	2(2.30%)	0.6207
NF1	8(4.02%)	4(2.08%)	0.3811	4(4.55%)	4(4.60%)	1.0000

ZRSR2	0	6(3.13%)	**0.0135**	1(1.14%)	2(2.30%)	0.6207
Prognosis *N*, %						
Death	132(63.77%)	99(47.83%)	**0.0015**	73(81.11%)	44(49.44%)	**< 0.0001**
Recurred/progresse	NA	NA		41(45.56%)	42(47.19%)	0.8813

BM, bone marrow; PB, peripheral blood; WBC, white blood cell; HGB, hemoglobin; PLT, platelet ; FAB, French–American–British classification systems; ELN, european leukemia network; *MLL*, myeloid/lymphoid or mixed–lineage leukemia; *EVI1*, Ecotropic Viral Integration Site 1; *PML*, promyelocytic leukemia protein; *RARA*, retinoic acid receptor alpha; *CBFB*, core–binding factor subunit beta; *MYH11*, myosin heavy chain 11; *RUNX1*, runt related transcription factor 1; *RUNX1T1*, runt related transcription factor 1 translocated to 1; *NPM1*, nucleophosmin 1; *FLT3*, fms related receptor tyrosine kinase 3; ITD, internal tandem duplication; TKD, tyrosine kinase domain; *DNMT3A*, DNA methyltransferase 3 alpha; *NRAS*, neuroblastoma RAS viral (v–ras) oncogen; *TET2*, ten–eleven translocation 2; *CEBPA*, CCAAT enhancer binding protein alpha; *IDH*, Isocitrate Dehydrogenase (NADP(+)); *TP53*, tumor protein P53; *WT1*, wilms tumor 1; *PTPN11*, protein tyrosine phosphatase non-receptor type 11; *KIT*, proto–oncogene tyrosine–protein kinase kit; *U2AF1*, U2 small nuclear RNA auxiliary factor 1; *KRAS*, kirsten rat sarcoma viral proto–oncogene; *SMC1A*, structural maintenance of chromosomes 1A; *BCOR*, BCL6 corepressor; *SF3B1*, splicing factor 3b subunit 1; *GATA*, GATA binding protein; *STAG2*, STAG2 cohesin complex component; *EZH2*, enhancer Of zeste 2 polycomb repressive complex 2 Subunit; *PHF6*, PHD Finger Protein 6; *SMC3*, structural maintenance of chromosomes 3; *RAD21*,

RAD21 cohesin complex component; *JAK*, janus kinase; *BCORL1*, BCL6 corepressor Like 1; *CBL*, Cbl proto–oncogene; *CSF3R*, colony stimulating factor 3 receptor; *NF1*, neurofibromin 1; *ZRSR2*, zinc finger CCCH–Type, RNA binding motif and serine/arginine rich 2; CR, complete remission.

The *p* value less than 0.05 was bold.

Supplementary Table S2. Multivariate Cox analysis of the three–PSMs score, genetic abnormalities, and clinical parameters in BeatAML2.0.

Variables	ELN2017-based OS			ELN2022-based OS		
	Total	HR(95%CI)	*p* value	Total	HR(95%CI)	*p* value
three–PSMs Score	*N* = 414	1.753 (1.059 - 2.900)	**0.029**	*N* = 414	1.824 (1.121 - 2.967)	**0.015**
Age	*N* = 414	1.036 (1.024 - 1.049)	**< 0.001**	*N* = 414	1.037 (1.025 - 1.049)	**< 0.001**
Gender	*N* = 414	1.306 (0.936 - 1.821)	0.117	*N* = 414	0.800 (0.580 - 1.103)	0.174
WBC	*N* = 353	1.004 (1.001 - 1.006)	**0.002**	*N* = 353	1.004 (1.001 - 1.006)	**0.002**
ELN	*N* = 399					
Adverse	*N* = 166	Reference	/	*N* = 194	Reference	/
Intermediate	*N* = 88	1.597 (0.956 - 2.668)	0.074	*N* = 114	0.848 (0.520 - 1.384)	0.510
Favorable	*N* = 145	0.529 (0.296 - 0.944)	**0.031**	*N* = 86	0.377 (0.206 - 0.690)	**0.002**
NPM1 mutation	*N* = 414	1.892 (1.198 - 2.989)	**0.006**	*N* = 414	1.550 (0.997 - 2.409)	0.052
RUNX1 mutation	*N* = 414	1.647 (0.989 - 2.743)	0.055	*N* = 414	1.352 (0.844 - 2.167)	0.210
TP53 mutation	*N* = 414	1.851 (1.014 - 3.377)	**0.045**	*N* = 414	1.913 (1.049 - 3.487)	**0.034**
FLT3–TKD mutation	*N* = 414	1.590 (0.921 - 2.746)	0.096	*N* = 414	1.629 (0.958 - 2.772)	0.072

JAK2 mutation	*N* = 395	0.597 (0.228 - 1.562)	0.293	*N* = 395	0.543 (0.209 - 1.415)	0.212
SRSF2 mutation	*N* = 395	0.943 (0.557 - 1.596)	0.827	*N* = 395	0.875 (0.520 - 1.470)	0.613
U2AF1 mutation	*N* = 395	1.726 (0.901 - 3.308)	0.100	*N* = 395	1.619 (0.842 - 3.113)	0.148
Complex Karyotype	*N* = 414	1.709 (0.984 - 2.967)	0.057	*N* = 414	1.359 (0.814 - 2.268)	0.240
—5/5q—	*N* = 414	0.926 (0.470 - 1.825)	0.825	*N* = 414	0.837 (0.435 - 1.613)	0.596
Trisomy 8	*N* = 414	1.413 (0.860 - 2.321)	0.172	*N* = 414	1.492 (0.906 - 2.458)	0.116
—7/7q—	*N* = 414	1.551 (0.830 - 2.898)	0.169	*N* = 414	1.779 (0.951 - 3.330)	0.072

PSMs, proteasome family members; OS, overall survival; HR, hazard ratio; WBC, white blood cell; ELN2017, European LeukemiaNet 2017; *NPM1*, Nucleophosmin 1; *RUNX1*, RUNX Family Transcription Factor 1; *TP53*, Tumor Protein P53; *FLT3–TKD*, Fms Related Receptor Tyrosine Kinase 3–tyrosine kinase domain; *JAK2*, Janus Kinase 2; *SRSF2*, Serine And Arginine Rich Splicing Factor 2; *U2AF1*, U2 Small Nuclear RNA Auxiliary Factor 1. The *p* value less than 0.05 was bold.

Supplementary Table S3. Multivariate Cox analysis of the three–PSMs score, genetic abnormalities, and clinical parameters in TCGA.

Variables	Total	OS	
		HR(95%CI)	*p* value
three–PSMs Score	*N* = 179	1.016 (1.005 - 1.026)	**0.003**
Age	*N* = 179	1.041 (1.025 - 1.057)	**< 0.001**
Gender	*N* = 179	0.753 (0.516 - 1.099)	0.141
WBC	*N* = 179	1.006 (1.002 - 1.011)	**0.003**
Cytogenetic risk	*N* = 176		
Good	*N* = 33	Reference	/
Intermediate	*N* = 101	2.427 (1.124 - 5.240)	**0.024**
Poor	*N* = 42	4.978 (2.054 - 12.065)	**0.001**
PML::RARA	*N* = 179	0.721 (0.236 - 2.209)	0.567
—7/7q—	*N* = 179	0.645 (0.297 - 1.401)	0.268

PSMs, proteasome family members; TCGA, the cancer genome atlas; OS, overall survival; HR, hazard ratio; WBC, white blood cell; *PML*, PML Nuclear Body Scaffold; *RARA*, Retinoic Acid Receptor Alpha.

The *p* value less than 0.05 was bold.

Supplementary Table S4. Main findings concerning our developed three–PSMs risk system in BeatAML2.0, TCGA, and GEO datasets.

Main Findings	BeatAML2.0	TCGA	GSE37642	GSE12417
The high expression of *PSMB8* and *PSMG1* and low expression of *PSMG4* were independent prognostic factors that were most strikingly associated with poor OS prognosis.	Yes	NA	NA	NA
The high three–PSMs risk scores correlated with high proportions of AML–M2 subtype, worse ELN stratification, no complete remission after induction therapies, and death.	Yes	NA	NA	NA
The high three–PSMs risk were linked to significantly poor OS prognosis.	Yes	Yes	Yes	Yes
The combined three–PSMs and ELN2017 or ELN2022 risk system had superior DCA and Harrell C–index than that which the corresponding ELN classification alone did, respectively.	Yes	Yes	NA	NA
The high three–PSMs risk group had higher frequencies of adverse genetic abnormalities.	*U2AF1*, *SRSF2*, *RUNX1*, and *TP53* mutations and *−7/7q−*, *−5/5q−*, *+8*, and *complex karyotype*	*-7/7q-*	NA	NA
The three–PSMs model was helpful in improving the prediction ability of ELN 2017 and 2022 classifications for OS.	Yes	Yes	NA	NA

The three–PSMs model could capture gene expression signatures related to leukemogenesis and chemotherapy resistance of AML.	Yes	Yes	Yes	Yes
The high–three–PSMs–risk AML cells were possibly sensitive and resistant to three (Lovastatin, AGI-5198, and SU11274) and two (Motesanib [AMG–706] and Cediranib [AZD2171]) compounds.	Yes	NA	NA	NA

Supplementary Table S5. Lists of potential limitations of our study which should be improved in the future.

Potential limitations	Possible influence	Improvement approach
Single origin and small size of enrolled samples in the validation cohort	Some biases in factors that influenced the patients' clinical outcomes, for example, race and socioeconomic status	Adding real–world and distinct races-belonging samples
Absence of experimental validations concerning key findings	The study only as a relative evaluation	Detection the three PSMs expression level or the effect of candidate compounds in the leukemia cell lines or fresh patient samples
Undetermination the validity of the ELN2022 stratification between different parameters	Not easily distinguish the difference in performance of ELN 2017 and 2022 risk system	Adding analysis using multiple origins and larger–scale patient datasets

Supplementary Figures

Figure S1

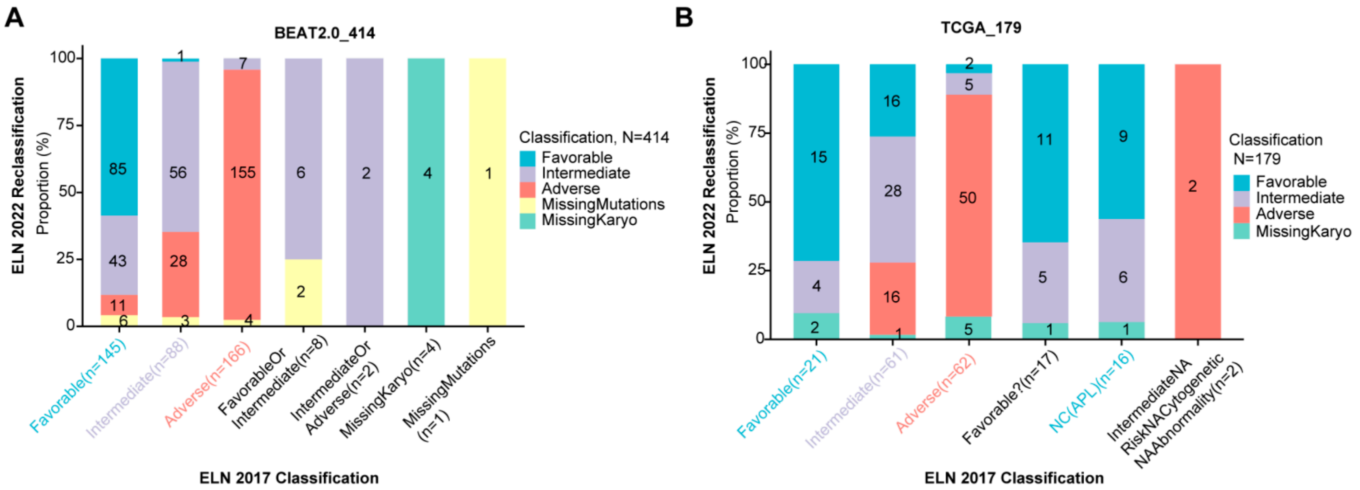

Supplementary Figure S1. The distribution of the ELN2022 prognostic scheme by reclassifying the 2017 version. The proportion of distinct ELN 2022 risk stratifications was compared to those of ELN 2017 in 414 patients from (**A**) BeatAML2.0 and and 179 patients from the (**B**) TCGA cohort, respectively.

Figure S2

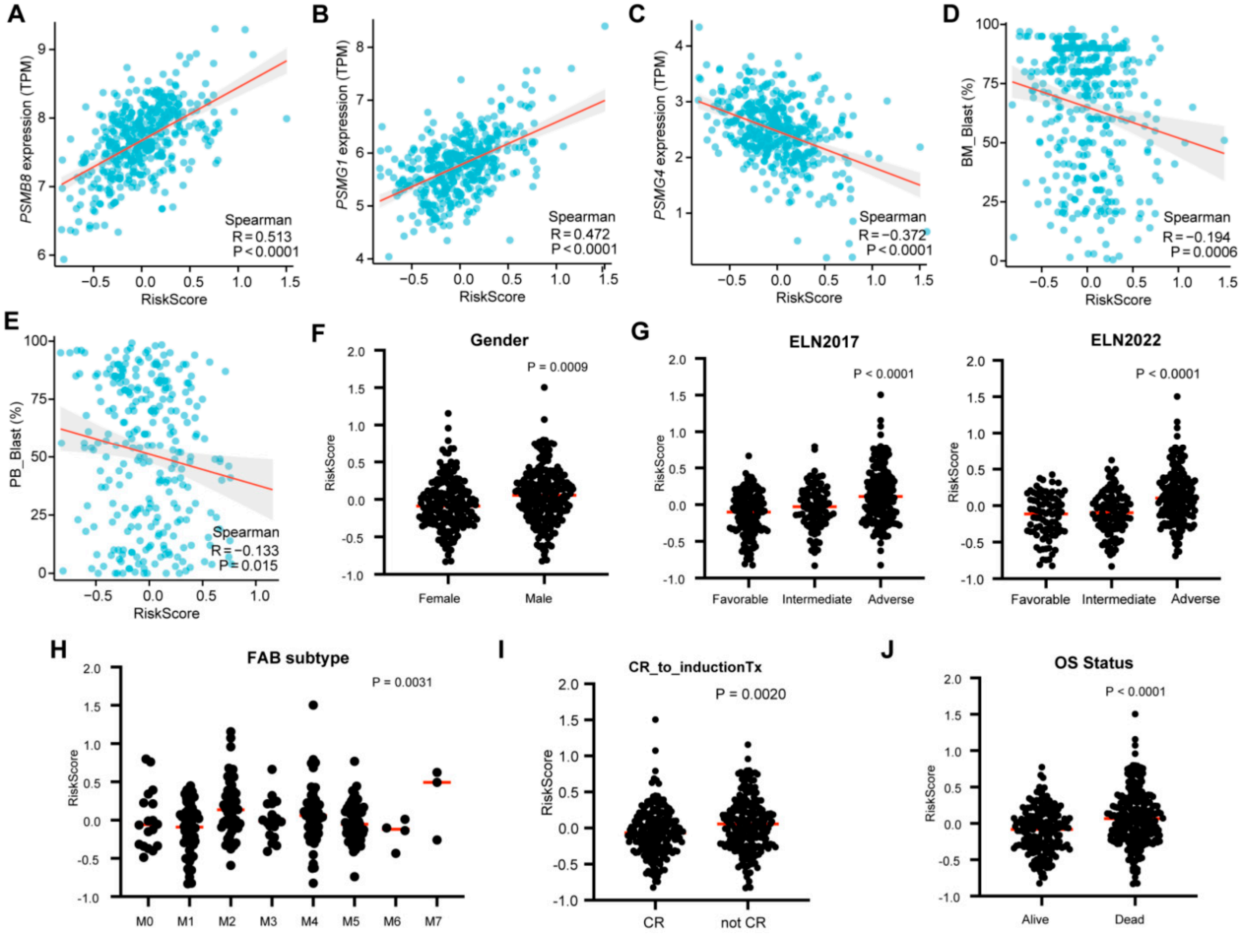

Supplementary Figure S2. The relationship of the three–PSMs score with gene expression and clinical parameters in AML. Scatter plots generated from Spearman rank correlation analysis demonstrated significant associations between the expression of *PSMB8* (**A**), *PSMG1* (**B**), and *PSMG4* (**C**) and the proportion of leukemia blast cells in both bone marrow (**D**) and peripheral blood (**E**), as well as with the three–PSMs score. Additionally, Mann-Whitney and Ordinary one – way ANOVA test were conducted to compare the risk score differences across various groups, including gender (**F**), European LeukemiaNet (ELN) 2017 and 2022 stratification (**G**), French – American – British classification systems (FAB) subtype (**H**), complete remission status following induction chemotherapy (**I**), and survival outcome (**J**). A *p*–value less than 0.05 was as having statistical significance.

Figure S3

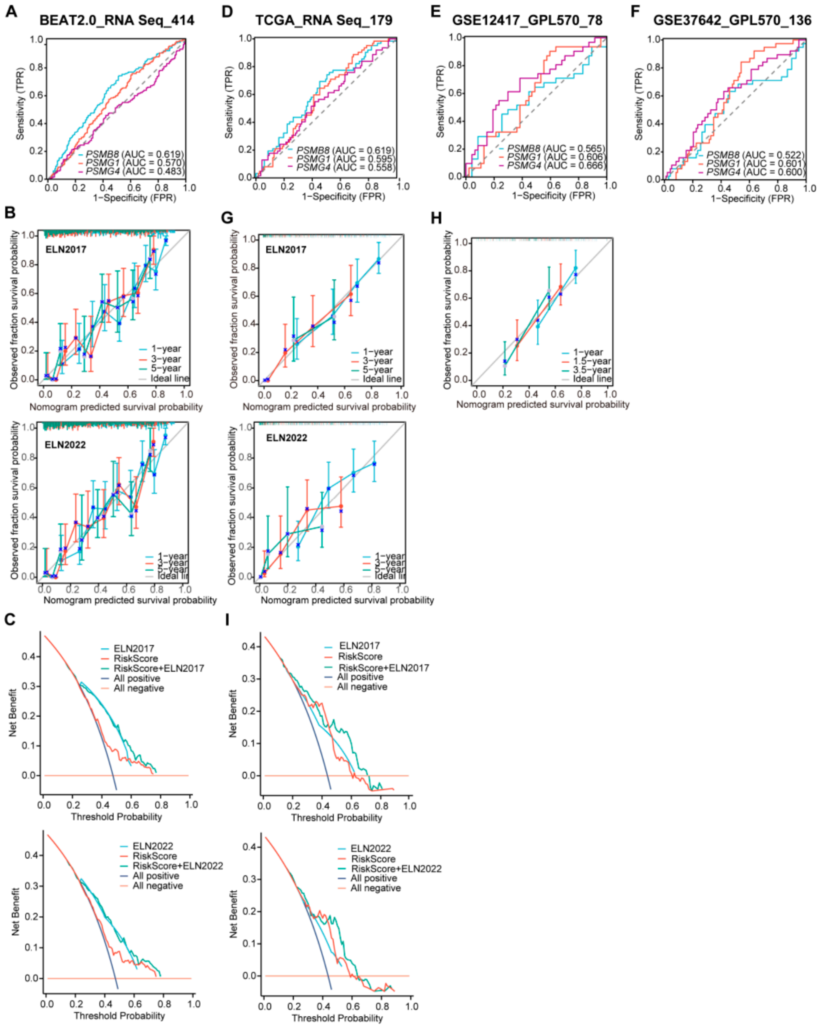

Supplementary Figure S3. Estimation of the prediction capability of the three–PSMs model for AML. (**A and D – F**) The diagnostic receiver operating characteristic (ROC) analysis assessed the area under curves (AUCs) for *PSMB8*, *PSMG1*, and *PSMG4* within the established three–PSMs model across the training and validation datasets. (**B, G, and H**) The calibration graph generated from the nomogram analysis, which included clinical factors (for example, either ELN2017 or 2022 classification) and the three–PSMs score, exhibited the consistency level between the predicted and observed survival probabilities for AML in BeatAML2.0 and the two validation genesets. (**C and I**) The decision curve analysis (DCA) compared the predictive benefits of ELN2017 and 2022 classification, the three–PSMs score, and their combined model in forecasting AML prognosis in the BeatAML2.0 and TCGA.

Figure S4

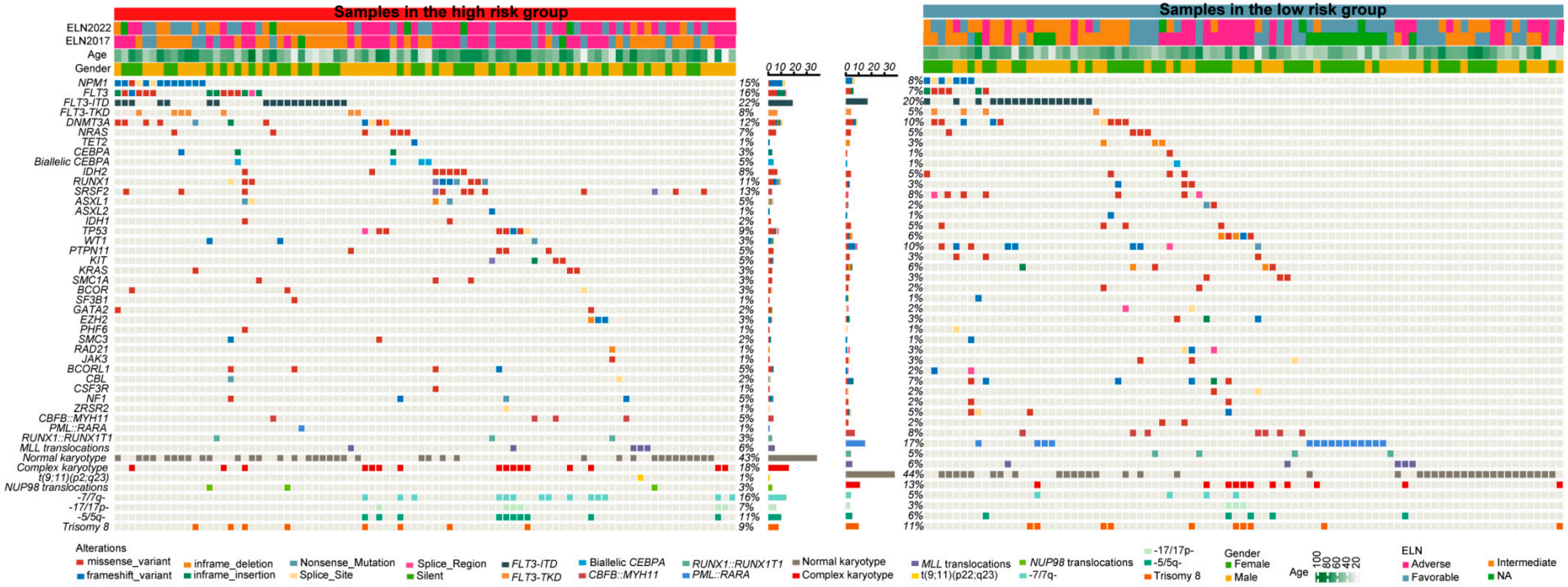

Supplementary Figure S4. The three–PSMs model–based genetic abnormalities landscape in TCGA. The heatmap showed the frequent molecular and cytogenetic variations and clinical indicators between the three–PSMs high and low–risk groups in the TCGA cohort.

Figure S5

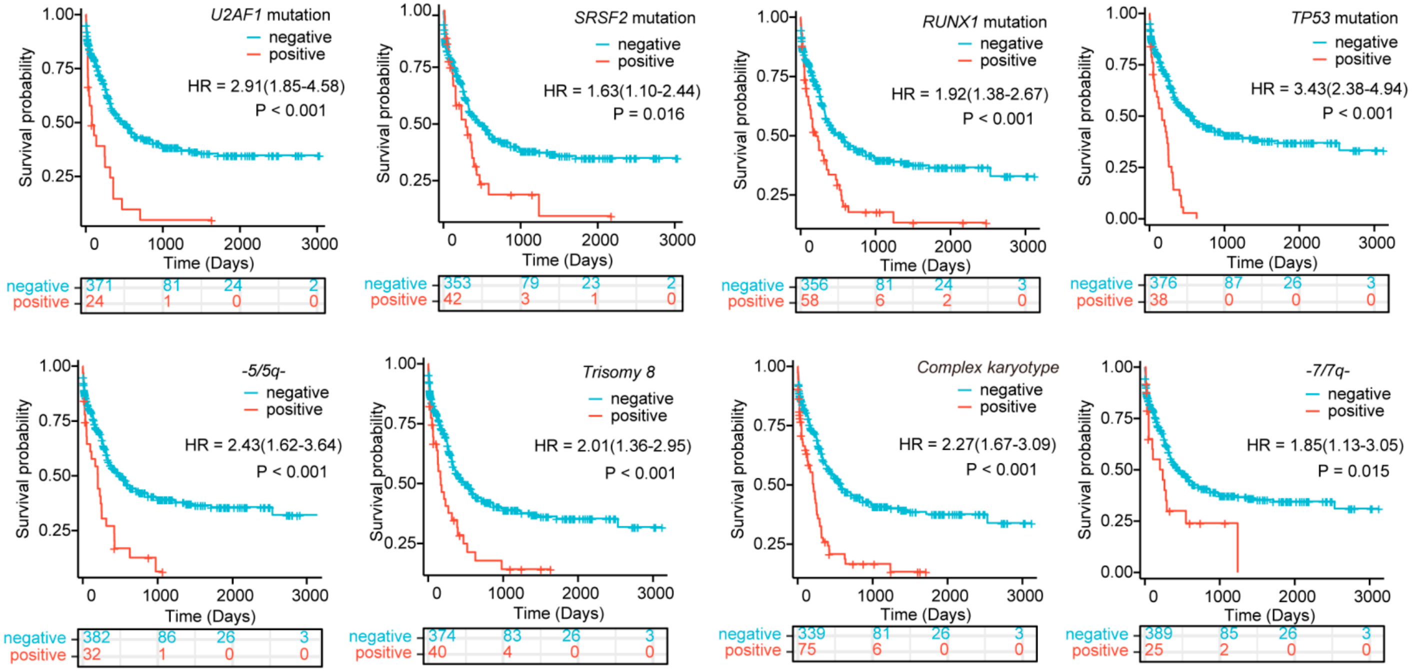

Supplementary Figure S5. The overall survival analysis of specific genetic alterations in the BeatAML2.0 cohorts. The K－M plots assessed the prognosis significance of mutations in *U2AF1*, *SRSF2*, *RUNX1*, and *TP53,* as well as the cytogenetic abnormalities －*5/5q*－, trisomy 8, complex karyotype, and －*7/7q*－ on overall survival (OS) of AML in the BeatAML2.0 dataset.

Figure S6

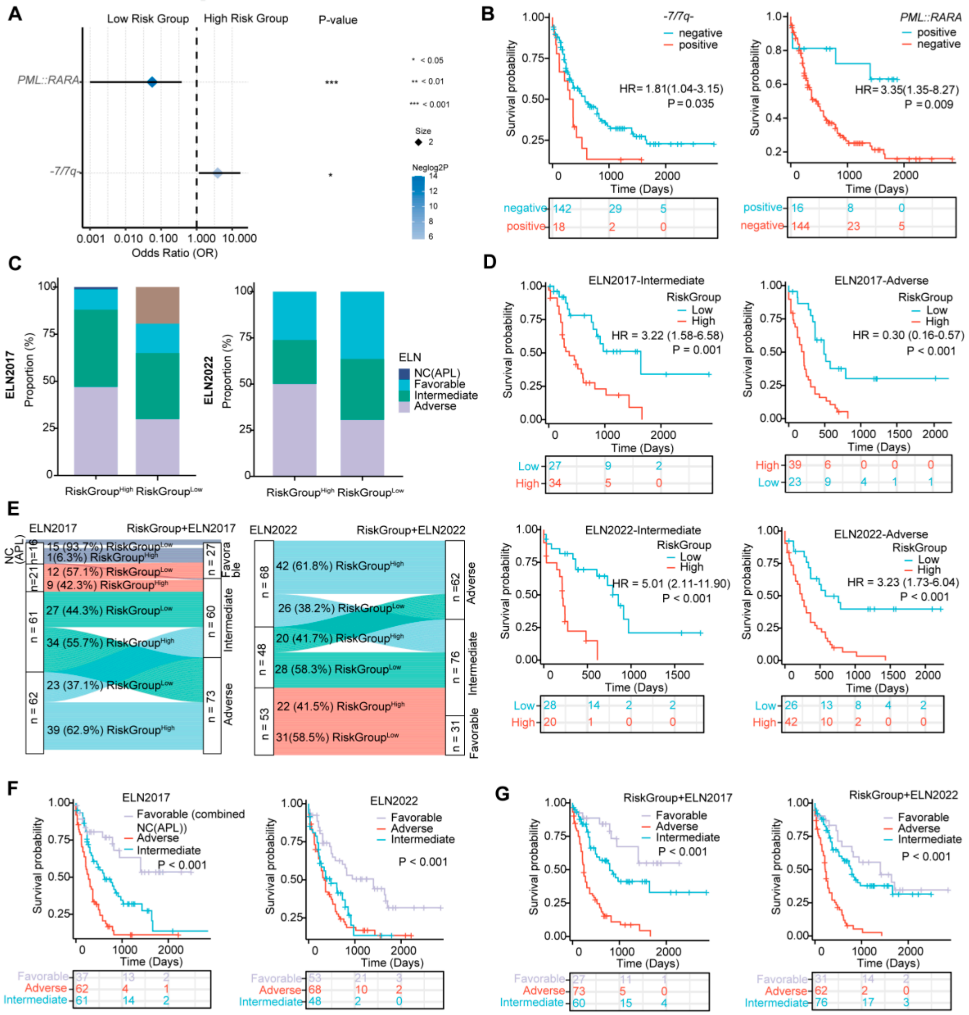

Supplementary Figure S6. The optimization of the three–PSMs model for ELN stratification in TCGA. (**A**) The forest plot outputted the genetic alterations with significantly different frequencies between the high and low–risk groups. (**B**) The K–M plots assessed the impact of *PML::RARA* and *−7/7q−* on OS in AML. (**C**) The histogram displayed the differential distribution of ELN2017 and 2022 stratifications within the high and low–risk cohorts. (**D**) The K–M curves highlighted significant differences in OS between high and low-three – PSMs – risk arms among the ELN2017 and 2022-intermediate and adverse subgroups. (**E**) The Sankey diagram depicted the reclassification flow of ELN2017 and 2022 by integrating the three–PSMs model, notably treating ELN2017–NC(APL, acute promyelocytic leukemia) as ELN2017–favorable. The K–M analysis estimated OS across ELN2017 and 2022 (**F**), as well as the subgroups combined with the three–PSMs model (**G**).

Figure S7

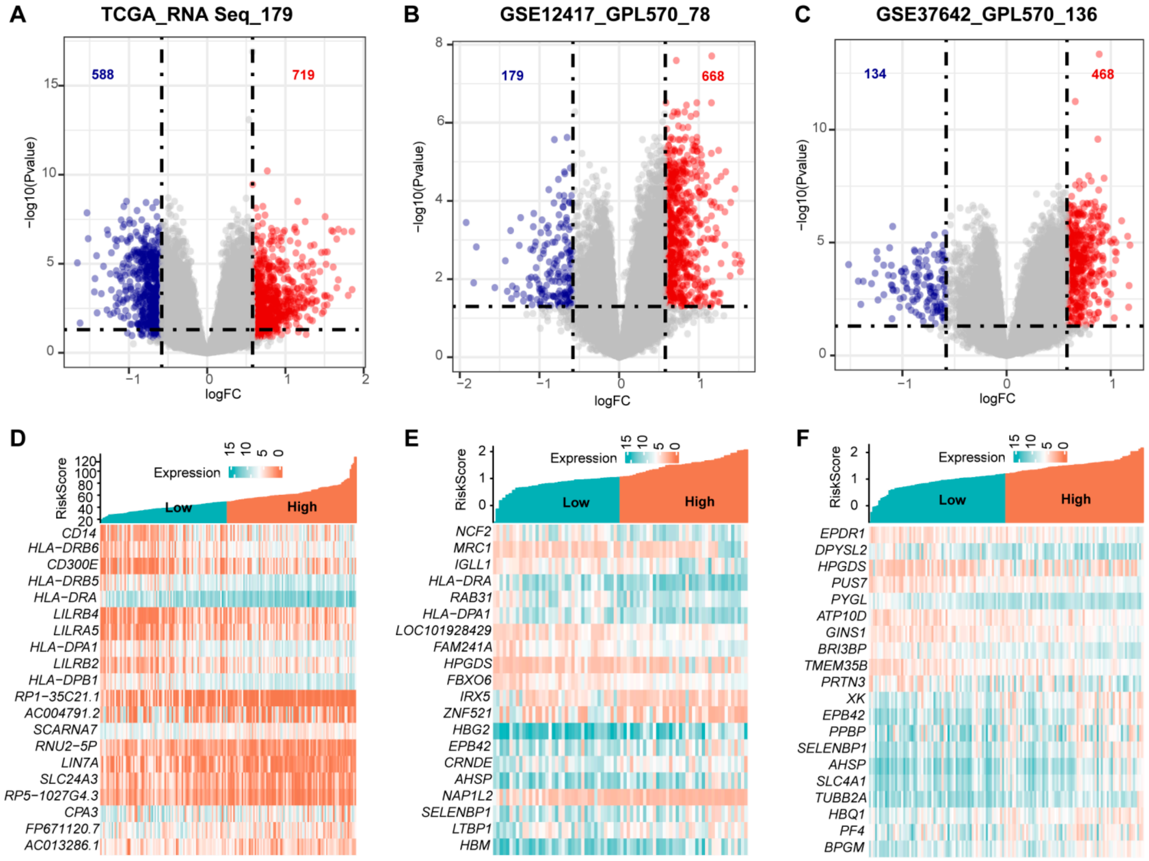

Supplementary Figure S7. Differential gene expression profile between the three–PSMs high and low–risk groups in the validation datasets. The volcano plots illustrated the differentially expressed genes defined by an absolute $\log_2$(fold change [FC]) > 0.58 and an adjusted *p*–value < 0.05 between the high and low–risk cohorts in TCGA (**A**), GSE12417 (**B**), and GSE37642 (**C**). (**D**–**F**) Heatmaps depicted the top 10 upregulated and deregulated genes among high and low – risk AML patients groups across these three datasets.

Figure S8

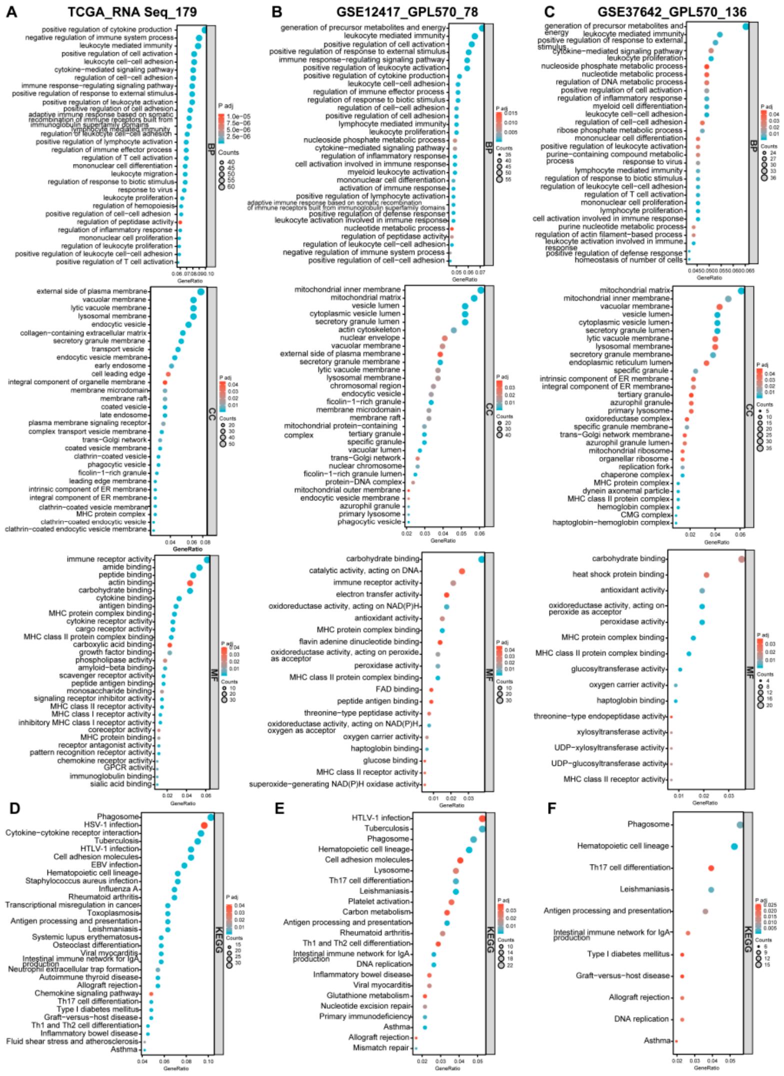

Supplementary Figure S8. The top-30 enrichment data of GO and KEGG analysis of the three−PSMs−based differential expressed genes. The bubble charts illustrated the enrichment results from gene ontology (GO) (**A**–**C**) and kyoto encyclopedia of genes and genomes (KEGG) (**D**–**F**) analyses, highlighting the top 30 categories ranked by gene counts based on differentially expressed genes between the high and low-three-PSMs–risk groups in the validates of TCGA, GSE12417, and GSE37642. The GO encompassed biological process (BP), cellular component (CC), and molecular function (MF). An adjusted *p*–value below 0.05 was considered statistically significant.
